# Carbon Mineralization in CO_2_–Seawater–Basalt Systems: Reactive Transport Dynamics and Vesicular Pore Architecture Controls

**DOI:** 10.1021/acs.langmuir.6c00958

**Published:** 2026-04-15

**Authors:** Mohammad Nooraiepour, Mohammad Masoudi, Helge Hellevang

**Affiliations:** † Environmental Geosciences, Department of Geosciences, 6305 University of Oslo, P.O. Box 1047 Blindern, Oslo 0316, Norway; ‡ 275243 SINTEF Industry, Applied Geoscience Department, S P Andersens Vei 15B, Trondheim 7465, Norway

## Abstract

Carbon mineralization in basaltic rocks offers a promising pathway for rapid, permanent CO_2_ storage, yet fundamental controls on reactive transport, precipitation patterns, and permeability evolution under seawater conditions remain poorly constrained. This study integrates flow-through column experiments at 80 °C with CO_2_-acidified seawater, geochemical modeling, and multiscale pore imaging (SEM-EDS and micro-CT) to elucidate mineralization dynamics in basaltic glass. Results demonstrate that carbonate precipitation is nucleation-limited and kinetically controlled rather than thermodynamically driven or growth-dominated, forming discrete patchy accumulations despite sustained supersaturation. An order-of-magnitude reduction in flow rate (0.05 to 0.005 mL/min) was required to achieve visible precipitation, highlighting residence time as the primary control. Postexperiment characterization identified calcium carbonate and inferred smectite-like clays, with dissolution-induced surface roughening and localized precipitation evident across the column. Seawater chemistry further complicates mineralization kinetics and efficiency relative to freshwater systems. Micro-CT analysis of three vesicular basalt facies revealed low coordination numbers (modal = 2) and serial connectivity, contrasting sharply with higher-coordination sandstone networks. The connected porosity (1.3–32%) differs significantly from the total segmented porosity (18–42%), demonstrating that network topology, rather than total porosity, controls permeability. Pore-scale observations thus indicate that precipitation may render basalts inherently more vulnerable to permeability impairment from modest, distributed precipitation. We explore end-member precipitation-induced clogging scenarios in which small, distributed precipitates cause disproportionately severe permeability loss compared to large, isolated masses. These findings underscore the need for probabilistic reactive transport frameworks that incorporate realistic pore topologies and nucleation barriers, which are fundamentally different from conventional CCS in sedimentary reservoirs, to improve predictions of injectivity and long-term carbon mineralization performance in mafic formations.

## Graphical abstract

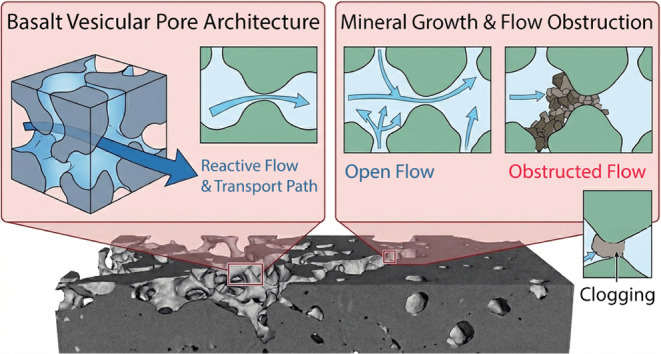

## 1 Introduction

The escalating climate crisis, driven primarily by human-generated CO_2_ emissions, underscores the urgent need for scalable mitigation solutions. Atmospheric CO_2_ concentrations have surged to unprecedented levels, driving rising global temperatures, ocean acidification, and extreme weather events.^1,2^ To limit global warming to 1.5–2 °C above preindustrial levels, as outlined in the Paris Agreement,^3^ it is imperative to not only reduce CO_2_ emissions but also deploy technologies for capturing and storing CO_2_ at scale. Carbon capture and storage (CCS) and geological carbon storage (GCS) have emerged as viable strategies for safely and permanently sequestering large volumes of CO_2_.^4–6^

Saline aquifers are the primary choice for geological CO_2_ sequestration due to their large storage capacity, widespread availability, and proximity to emission sources.^4,7,8^ However, long-term storage security in saline aquifers relies heavily on caprock integrity to prevent buoyant CO_2_ migration to the surface,^9,10^ as these systems primarily target structural/stratigraphic and residual trapping mechanisms. In contrast, GCS in mafic and ultramafic rocks offers a promising alternative with the added benefit of CO_2_ mineralization, achieving rapid mineral trapping.^11–14^ Basaltic rocks, rich in divalent cations, such as calcium (Ca^2+^), magnesium (Mg^2+^), and iron (Fe^2+^) in silicate minerals, react with CO_2_ to form stable carbonate minerals. The mineral trapping mechanism represents the most secure form of carbon storage.

The potential of mafic and ultramafic formations for carbon mineralization has been demonstrated through several pioneering projects worldwide, including the CarbFix project in Iceland^11,15^ the Wallula pilot project in Washington, USA^16,17^ and emerging initiatives such as CO_2_Lock,^18^ CarbonStonev,^19^ and 44.01.^20^ The CarbFix project leverages CO_2_ dissolution in freshwater to form carbonic acid, which reacts with basaltic rocks to precipitate carbonate minerals, with a reported 95% mineralization rate within 2 years at 20–50 °C.^11,15^ However, this approach requires substantial freshwater resources—approximately 22 tons per ton of CO_2_ at 30 bar and 20 °C—raising scalability concerns and potential conflicts with water resources. In contrast, the Wallula project directly injects supercritical CO_2_ (scCO_2_) into basaltic formations, relying on caprock integrity for containment, with hydrological modeling suggesting up to 60% mineralization within 2 years.^14,16,17^

These projects demonstrate the feasibility of in situ mineral carbonation in mafic and ultramafic rocks under localized conditions. However, global scalability requires expanding field sites and addressing key challenges: optimizing CO_2_ injection rates, ensuring consistent long-term fluid movement within storage reservoirs, and enhancing dissolution–precipitation rates. Despite the successes of pilot projects, deriving detailed hydrological, chemical, and mechanical parameters from field observations alone remains challenging. Critical gaps persist in scaling this technology globally. First, reported mineralization rates must be sustained at megaton-scale injection rates, requiring a deeper understanding of geochemical and reactive transport processes under field conditions. Second, seawater or in situ saline water must replace freshwater to enhance applicability, particularly for offshore basalt deposits. These offshore basaltic formations offer immense theoretical storage potential—up to 100,000 gigatonnes of CO_2_, exceeding 2,000 times current annual global emissions. Additional critical questions requiring urgent attention include: (i) evaluating scCO_2_ injection regarding reactive transport and containment integrity, which requires secure caprocks and extended retention times for complete mineralization; (ii) determining realistic time scales for mineral dissolution and precipitation processes to provide industrial-scale timeframes for mineral trapping; and (iii) identifying reactive surface areas and reaction rates in fractures (where advection dominates) versus pores and matrices (where diffusion prevails) to enable stochastic simulation of reactive flow scenarios.

Despite numerous reviews on carbon mineralization,^14,21–28^ the geochemical interactions and reactive transport dynamics of CO_2_-rich seawater in basalts remain poorly understood. To address knowledge gaps regarding seawater use as the aqueous phase under reactive flow conditions, this study integrates laboratory experiments with geochemical fluid-rock interaction simulations to assess CO_2_ mineralization dynamics. We focus on three key aspects: (1) the potential for rapid CO_2_ mineralization using seawater under reactive transport conditions, (2) the effect of residence time on mineralization through advection velocity variations, and (3) the pore space architecture and characteristics of vesicular basalt reservoirs.

## 2 Challenges of Carbon Mineralization in Basalts Using Seawater

Carbon mineralization in basaltic rocks using CO_2_-charged seawater is complicated by competing reactions that occur under varying thermodynamic and geochemical conditions. The substitution of seawater for freshwater introduces additional challenges, affecting carbonate nucleation and growth, and creating substantial uncertainties in reaction pathways and efficiency due to heterogeneous mineral-fluid interactions.

Experimental studies reveal that interactions of CO_2_-charged seawater with basaltic rocks are highly temperature- and composition-dependent. Carbon mineralization efficiency is maximized at 90–150 °C, where silicate dissolution and carbonate precipitation rates are optimally balanced. Lower temperatures (<50 °C) result in slower reaction kinetics, while temperatures above 200 °C cause retrograde solubility and formation of competing phases, such as zeolites, decreasing yields. Rosenbauer et al.^29^ demonstrated ferroan magnesite formation at 100 °C achieving 8–26% CO_2_ mineralization, while Shibuya et al.^30^ observed calcite as the dominant precipitate at 250–350 °C, reducing dissolved CO_2_ concentrations by 75–100%.

The high Mg^2+^ content in seawater competes with Ca^2+^ for carbonate incorporation, often forming mixed Ca–Mg carbonates with lower stability than pure calcite or magnesite.^31–33^ The presence of sulfate catalyzes the precipitation of anhydrite and gypsum (CaSO_4_), thereby reducing porosity and sequestering essential Ca^2+^ ions. Rigopoulos et al.^34^ demonstrated that removing sulfate from artificial seawater minimized anhydrite interference, increasing carbonate yield by 30%. However, replicating these conditions in natural seawater systems adds operational complexity, particularly for offshore implementations.

Kinetic limitations arise from the formation of metastable byproducts. Voigt et al.^35^ showed that high CO_2_ partial pressure (16 bar) favors magnesite over Mg-rich clays in submarine basalts, mineralizing 20% of CO_2_ in 5 months. At lower pressures, clay formation consumes Mg^2+^ and stalls carbonate growth. Wolff-Boenisch and Galeczka^36^ demonstrated that induced Ca/Mg-carbonate precipitation requires artificially elevated carbonate saturation, which is rarely achieved in natural reservoirs. In the absence of engineered supersaturation, secondary minerals (e.g., clays and zeolites) may sequester key cations, which raises the nucleation energy barrier and renders nucleation less energetically favorable. Without engineered supersaturation, secondary minerals such as clays and zeolites sequester crucial cations, reducing free Mg^2+^ (and Ca^2+^) concentrations and thus lowering carbonate supersaturation (Ω). This increases the nucleation energy barrier for magnesite, rendering spontaneous carbonate formation kinetically unfavorable.

The efficiency of seawater-based systems depends critically on optimizing temperature and pH. Lower temperatures and acidic conditions inhibit carbonate precipitation or favor noncarbonate phases such as smectites.^37^ Robust carbonate formation occurs at elevated temperatures and near-neutral pH (7.6–8.7), emphasizing the importance of optimizing reaction conditions.

While numerous batch-type laboratory studies^35,38–42^ have advanced the understanding of basalt–seawater–CO_2_ interactions, translating these findings to field-scale applications remains challenging. These investigations underscore the need for further geochemical and reactive transport studies, complemented by the characterization of secondary minerals, particularly in submarine environments. Extensive research is necessary to optimize reaction conditions for seawater-based carbon mineralization in basaltic systems, particularly at lower temperatures and near-neutral pH, in order to elucidate the complex balance that governs successful outcomes.

## 3 Materials and Methods

### 3.1 Flow-Through Column Reactor Experiments

Flow-through reactive transport experiments were conducted by using a custom-designed glass tube reactor (40 cm length, 14 mm inner diameter). The reactor was filled and carefully packed with two distinct zones: crushed calcium carbonate grains (500–1000 μm in size) in the initial 4 cm section to provide dissolved Ca^2+^ and pH buffering after reacting with injected CO_2_-acidified seawater, and basaltic glass occupying the remaining 36 cm as the primary substrate for carbonate nucleation and growth.

The custom-designed transparent glass tube reactor was chosen over a traditional core flood to enable a sufficiently long flow path (40 cm) for the development of observable chemical gradients, real-time visual monitoring of precipitation dynamics due to optical transparency, and reproducible uniform packing of crushed grains with controlled flow distribution at the inlet and outlet. These features were critical for distinguishing deterministic supersaturation-driven outcomes from probabilistic nucleation-controlled patterns and for facilitating detailed postmortem imaging analysis.

Basaltic glass from Stapafell Mountain, Reykjanes Peninsula, Iceland, served as the primary reactive substrate for the column experiments. This tholeiitic basalt, with a composition comparable to mid-ocean ridge basalt (MORB) (Table 1), exhibits a divalent cation oxide content (CaO + MgO + Fe_2_O_3_ = 23.8 wt %) optimal for carbon mineralization.

**Table 1:** Chemical Composition (Wt%) of Stapafell Basaltic Glass, Determined by XRF Analysis^a^

	SiO_2_	TiO_2_	Al_2_O_3_	Fe_2_O_3_	MnO	MgO	CaO	Na_2_O	K_2_O	P_2_O_5_	Cr_2_O_3_	ZnO	SO_3_
wt %	47.282	1.487	14.657	12.148	0.213	10.079	11.438	1.756	0.273	0.206	0.109	0.013	0.111

a Fe_2_O_3_ represents total iron, including Fe^2+^ and Fe^3+^.

The crushed calcium carbonate and basaltic grains were ultrasonically cleaned in Milli-Q ultrapure deionized water and subsequently dried at ambient temperature before being added to the reactor. The basaltic glass, sourced from Stapafell (Reykjanes Peninsula, Iceland), was selected for its rapid reactivity, homogeneous chemical composition, and dark coloration, which facilitates the identification of secondary carbonate phases.

Seawater (∼1 L) was collected from Oslo Fjord, filtered through 0.45 μm Millipore filters, and used as the base fluid due to its chemical similarity to average North Sea seawater. The seawater was acidified by CO_2_ dissolution at 4 MPa in a pressurized fluid accumulator housed within a forced-convection benchtop oven. The equilibrated CO_2_-charged seawater was injected using a dual-piston ISCO pump at two controlled flow rates: 0.05 and 0.005 mL/min, enabling the systematic evaluation of residence time effects on mineral precipitation kinetics. The column had a total volume of 61.6 mL and a pore volume of approximately 25 mL. At the higher flow rate of 0.05 mL/min, this corresponds to ∼1 PV exchanged every ∼8.2 h, with a Darcy velocity of 5.4 × 10^–6^ m/s (0.032 cm/min) and an interstitial (pore) velocity of 1.4 × 10^–5^ m/s (0.081 cm/min). At the lower flow rate of 0.005 mL/min, ∼1 PV was exchanged every ∼82 h, with a Darcy velocity of 5.4 × 10^–7^ m/s (0.0032 cm/min) and a pore velocity of 1.4 × 10^–6^ m/s (0.0081 cm/min). Independent replicate experiments (n = 2 per flow rate) were performed under identical conditions (same seawater batch, temperature, pressure, and substrate preparation) to assess reproducibility and variability in reactive transport and mineral precipitation outcomes. All replicates showed consistent behavior: no macroscopic carbonate precipitation at 0.05 mL/min and patchy precipitation at 0.005 mL/min. This flow-rate variation provided residence times that enabled the assessment of the impacts of advection velocity on carbonate formation.

Experiments were conducted at 80 °C with an injection pressure of approximately 1 MPa (automatically controlled by injection pumps) and atmospheric CO_2_ pressure at the outlet for 30 days. The experimental temperature (80 °C) and pressure (1 MPa) were chosen to achieve thermodynamically favorable conditions for carbonate precipitation while ensuring kinetically accessible growth rates within practical laboratory time scales. From classical nucleation theory and prior studies of carbonate formation in basaltic systems, 80 °C provides a practical “sweet spot” where precipitation and its consequences for fluid flow can be observed and characterized, whereas lower temperatures would have resulted in impractically slow reaction progress. The modest pressure was selected for operational simplicity and safety in the flow-through setup, while still enabling controlled CO_2_ dissolution, weak acid formation, and stable aqueous chemistry. Although these conditions do not replicate deep geothermal gradients, they effectively capture the essential thermodynamic and kinetic drivers relevant to understanding dissolution–precipitation dynamics and the extent of carbon mineralization in basaltic systems under seawater geochemistry.

Fluid samples were collected at discrete intervals (every 3 days) from the column inlet and continuously monitored at the outlet. The solution pH was measured immediately after sampling with a calibrated benchtop pH meter (Metrohm 780 pH/ion meter) to monitor geochemical evolution during the experiments. Detailed descriptions of the fluid injection system are provided in our prior studies.^43,44^

The inlet fluid was sourced from a large pressurized fluid transfer vessel (accumulator) housed within the forced-convection benchtop oven and continuously equilibrated by bubbling with a pressure-controlled CO_2_ stream to maintain a constant target pCO_2_. Given the large reservoir volume relative to the low daily withdrawal rate and active gas-phase equilibration, the inlet chemistry remained highly stable throughout the experiments. Discrete inlet sampling every 3 days was therefore sufficient to confirm this stability and detect any minor unintended drifts (e.g., from small temperature fluctuations or gas supply variations). More frequent sampling was unnecessary and would have risked introducing pressure perturbations during repeated sampling valve operations. In contrast, the outlet fluid underwent continuous reactive interaction with the basaltic glass column, leading to time-dependent changes in pH, alkalinity, major cations (Ca, Mg, Si, etc.), and saturation states driven by dissolution and precipitation. Continuous monitoring of the outlet was therefore essential to capture transient and evolving reactive transport dynamics, including nonsteady-state and flow-rate-dependent behaviors. The continuous outlet pH record also served as a real-time indicator of experimental stability and reactive system performance. All inlet measurements confirmed near-constant conditions over the experimental duration, while outlet trends showed clear time-dependent geochemical evolution.

Differential pressure across the column was continuously monitored by using pressure transducers installed at the injection pump, column inlet, and column outlet. The pressure drop (Δ*P*) was recorded every 1 min and used to calculate temporal changes in permeability via Darcy’s law. No statistically significant change in Δ*P* or permeability was observed over the test duration.

### 3.2 Sample Characterization

The mineralogical and elemental composition were identified and quantified using X-ray diffraction (XRD) and X-ray fluorescence (XRF), respectively, following the methodology described in our previous studies.^45,46^

Scanning electron microscopy (SEM) was used to examine the surface morphology and mineral growth on the substrates. Energy-dispersive X-ray spectroscopy (EDS) enabled chemical analysis and elemental mapping to identify mineral phases and their spatial distribution. A variable pressure Hitachi SU5000 FE-SEM instrument (Schottky FEG) with a Dual Bruker XFlash system was employed for imaging and spectroscopy. Samples were coated with a thin carbon layer to enhance image quality, improve chemical analysis accuracy, and prevent surface charging. Further details are given in Ref 47.

For pore space characterization, three distinct Icelandic basalt facies with varying degrees of vesicularity were analyzed, with two specimens per facies to capture heterogeneities and the range of variations. High-resolution X-ray microcomputed tomography (micro-CT) scans were acquired across spatial scales (1–10 μm voxel sizes), with the primary results in this manuscript based on 5 μm voxel-size scans to achieve a balanced compromise between the imaged domain size and the required resolution for pore-network extraction. Micro-CT imaging elucidates the three-dimensional pore structure of vesicular basalts, providing critical quantitative insights into porosity and pore connectivity that govern fluid flow and reactive transport. Tomographic data sets were processed using Dragonfly software (v2024.1) in conjunction with an in-house Python image processing pipeline. Phase segmentation was performed using a U-Net convolutional neural network trained on manually annotated slices as ground truth data (5 encoder-decoder levels, 64 initial convolutional filters, trained for 100 epochs using 64 × 64 × 1 pixel patches with categorical cross-entropy loss and Adadelta optimizer). The trained DL model was applied to the entire 3D volumes, with supplementary thresholding used to segment the pore volume in vesicular basalt. Both methods yielded consistent pore volumes (<1% variation). Image acquisition, processing, and segmentation followed the protocol described in detail in our previous works.^48,49^ Pore connectivity and pore-network feature extraction were performed using the OpenPNM framework,^50^ with connectivity defined by throat connections between pore bodies and no imposed minimum connected length scale beyond the voxel resolution. The extracted three-dimensional pore networks provided quantitative characterization of vertex (pore body) and edge (throat) properties, including connectivity, volume, diameter, and cross-sectional area distributions. PNM analysis of vesicular basalt samples was conducted as exploratory sensitivity studies rather than direct reproductions of the experimental columns, with precipitation patterns informed by site-specific preferences observed in the reactive transport experiments.

The three facies (low-, moderate-, and high-vesicularity) were selected to represent the principal range of vesicular textures expected in basaltic reservoirs, spanning end-member and intermediate porosity structures typical of certain lava flow facies. Within each facies, two specimens were chosen from visually distinct blocks collected several meters apart to capture intrafacies textural variability (e.g., differences in vesicle size, shape, and packing). While only two samples per facies (six total) were analyzed due to micro-CT scanning time and computational constraints of high-resolution PNM extraction, this approach revealed substantial heterogeneity, as reflected in the broad distributions of pore properties across the six samples. The sampling was designed to provide quantitative insight into topological controls on precipitation vulnerability rather than to provide an exhaustive statistical representation of all basaltic reservoirs, which exhibit wide variability in lava flow facies, alteration states, and fracture-dominated flow. The PNM-derived clogging thresholds are therefore presented as model-indicated order-of-magnitude guidance, conditional on the imaged vesicular topology, assumed clogging mode, and precipitation location preferences.

**Table 2:** Pore Network Modeling Statistics for Unreacted (Pristine) Vesicular Basalt Samples Derived from Micro-CT Segmentation^a^,^b^

Parameter	Unit	Sample A	Sample B	Sample C
*Vertex (Pore Body) Properties*				
Connectivity	–	3.2 ± 2.1	3.2 ± 2.6	5.6 ± 5.1
		(1.0–6.8)	(1.0–7.8)	(1.0–12.5)
Volume	mm^2^	0.01 ± 0.04	0.16 ± 0.48	0.06 ± 0.12
		(0.0001–0.0001)	(0.0001–0.51)	(0.00004–0.00004)
Surface area	mm^2^	0.09 ± 0.26	0.74 ± 1.52	0.17 ± 0.49
		(0.02–0.17)	(0.004–2.95)	(0.02–0.02)
Pore-specific SSA	mm^–1^	9.00	4.63	2.83
Bulk-specific SSA	mm^–1^	1.76	1.43	1.17
Inscribed diameter	mm	0.09 ± 0.06	0.24 ± 0.24	0.09 ± 0.06
		(0.05–0.16)	(0.03–0.73)	(0.05–0.15)
Equivalent diameter	mm	0.15 ± 0.09	0.35 ± 0.33	0.14 ± 0.10
		(0.07–0.28)	(0.06–0.97)	(0.04–0.26)
*Edge (Throat) Properties*				
Equivalent diameter	mm	0.08 ± 0.06	0.21 ± 0.24	0.08 ± 0.07
		(0.01–0.17)	(0.01–0.72)	(0.01–0.17)
Total length	mm	0.24 ± 0.13	0.64 ± 0.43	0.28 ± 0.21
		(0.11–0.50)	(0.17–1.49)	(0.11–0.71)
Cross-sectional area	mm^2^	0.01 ± 0.02	0.09 ± 0.23	0.02 ± 0.04
		(0.0001–0.02)	(0.0001–0.42)	(0.0001–0.0001)

a Connectivity represents the number of throats connected to each pore body in the entire volume. Inscribed diameter is the diameter of the largest sphere that fits within the pore. The equivalent diameter is the diameter of a sphere with equal volume. Cross-sectional area refers to the throat constriction area. High standard deviations (often exceeding means) for volume, surface area, and related parameters reflect the intrinsic multiscale heterogeneity of vesicular basalt pore architecture: wide-ranging vesicle sizes from sub-μm micropores to mm–cm macrovesicles, typically following heavy-tailed (e.g., power-law-like or multimodal) distributions due to primary degassing, coalescence, and secondary alteration. This skewness makes the arithmetic mean sensitive to rare large pores. The reported 5th–95th percentiles better represent typical values, while means are retained for comparability with literature PNM studies. Sample A connectivity values represent local clustering within isolated pore groups rather than network-scale connectivity.

b Vertex (pore body) and edge (throat) scalar values represent distributions across the extracted pore networks. Values are reported as mean ± standard deviation (indicating statistical spread), with 5th–95th percentile ranges in parentheses showing actual data bounds. Pore-specific surface area (SSA_pore_) is the ratio of the mean surface area to the mean pore volume. Bulk-specific surface area (SSA_bulk_) is SSA_pore_ × *ϕ*_pore_ × *ϕ*, using midpoint porosity for Samples A–C.

### 3.3 Geochemical Modeling

Geochemical modeling was performed using PHREEQC v3 ^51^ to simulate aqueous geochemical processes, including advective flow and dispersion, solute saturation states, pH evolution, CO_2_ pressure dynamics, and reaction progress. For calculations, the standard state is assumed to be the unit activity for pure minerals and H_2_O at specified temperature and pressure conditions, with a hypothetical 1 molal solution referenced to infinite dilution for all aqueous species.

Reactive transport simulations were implemented in PHREEQC to model column-averaged geochemical evolution and saturation-state trends along the flow path. While this deterministic continuum approach effectively captures bulk fluid chemistry and thermodynamic drivers, it inherently assumes perfect lateral mixing within cells and does not resolve pore-scale heterogeneities or probabilistic nucleation processes.

The CarbFix thermodynamic database^52^ was used for all calculations, which, in turn, is constructed from the core10.dat database derived from the llnl.dat database distributed with PHREEQC. This database selection ensures accurate thermodynamic predictions of mineral dissolution and precipitation reactions under the specific temperature, pressure, and compositional conditions of this study. The CarbFix database has been validated explicitly for basalt–CO_2_–water interactions, making it particularly suitable for modeling carbonate mineralization processes in mafic rock systems. The geochemical models served dual purposes: (1) interpreting experimental observations through thermodynamic equilibrium calculations and kinetic assessments, and (2) predicting long-term CO_2_ mineralization behavior in basaltic rocks under varying reactive transport conditions.

## 4 Results and Discussion

### 4.1 Basaltic Glass Composition and Characterization

The basaltic glass used in our study was sourced from Stapafell Mountain on the Reykjanes Peninsula, southwest Iceland. This material exhibits a tholeiitic composition, characterized by high silica (SiO_2_) and low sodium (Na_2_O) contents, with a fine-grained extrusive igneous texture.

XRD and XRF analyses confirmed that the composition of the Stapafell basaltic glass aligns closely with values reported in the literature.^53–56^ The major element composition, detailed in Table 1, is comparable to the mid-ocean ridge basalt (MORB).^53,57,58^ This basaltic material has been extensively characterized and utilized in prior kinetic and experimental studies.^35,37,53,55,56,59–62^

A detailed microscopic examination of the unreacted basaltic glass grains was conducted to establish the baseline surface morphology and compositional characteristics prior to reactive transport experiments. The pristine basaltic glass grains exhibit smooth, glassy surfaces at intermediate magnification (Figure 1b), with only natural cavities, vesicular structures, and conchoidal fractures visible at higher resolution (Figure 1c), confirming the absence of preexisting secondary minerals or significant weathering prior to the experiments.

**Figure 1:**
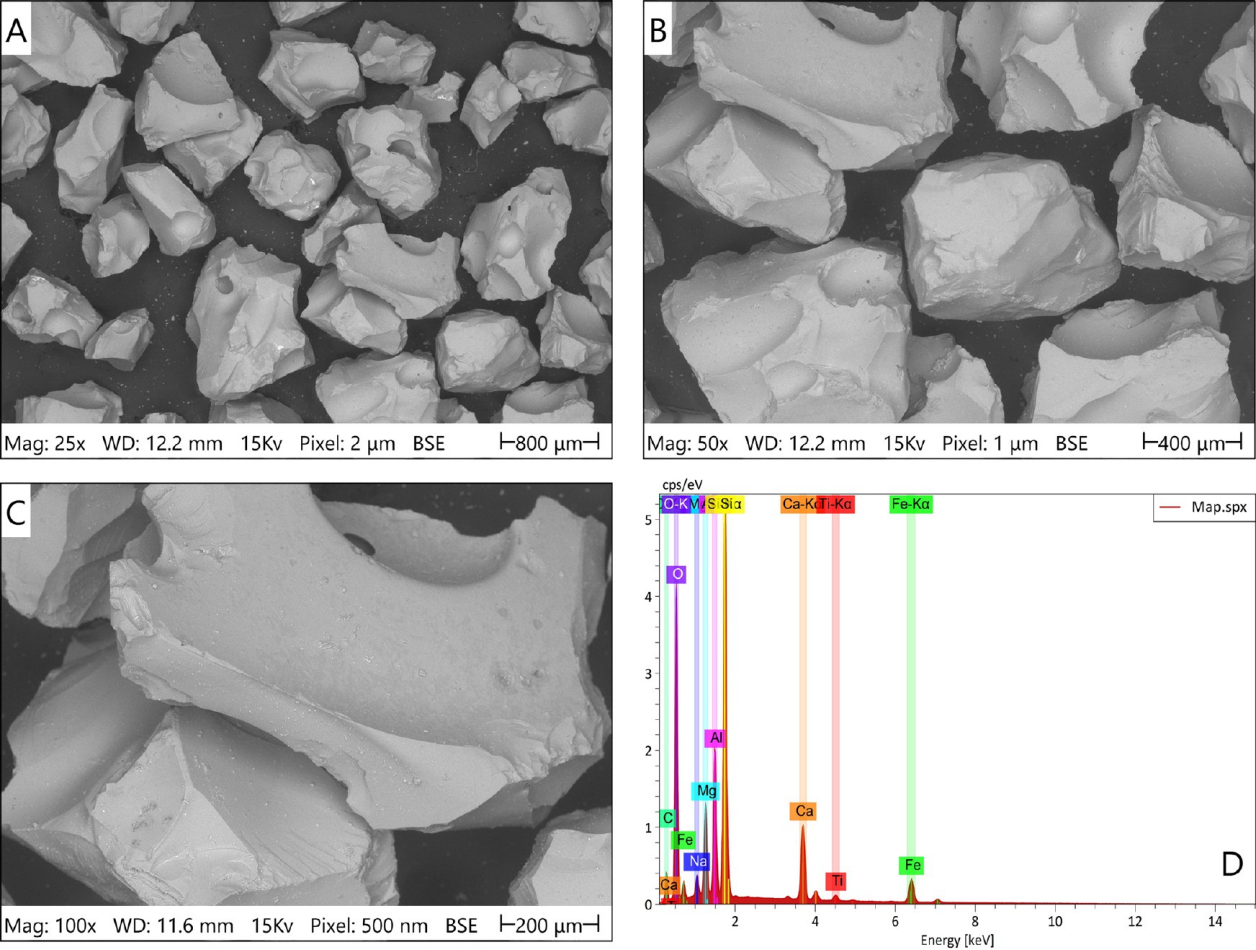
Multiscale SEM characterization of unreacted Stapafell basaltic glass grains used in flow-through experiments. (a) Low-magnification overview (25×, pixel size = 2 μm) showing grain size distribution and overall morphology. (b) Intermediate magnification (50×, pixel size = 1 μm) revealing characteristically smooth, glassy surfaces typical of rapidly quenched volcanic glass, with minimal pre-existing roughness. (c) High-magnification image (100×, pixel size = 0.5 μm) displaying natural surface features, including cavities, vesicular structures, and conchoidal fracture patterns, with no evidence of pre-existing secondary mineral phases or weathering products. (d) Representative EDS spectrum from pristine grain surfaces, confirming major elements (Si, Al, Fe, Ca, Mg, O) consistent with tholeiitic basalt composition and the availability of reactive divalent cations (Ca^2+^, Mg^2+^, Fe^2+^) for CO_2_ mineralization reactions. These observations establish the pristine, smooth baseline morphology of the substrate prior to reactive transport experiments.

Figure 1 presents multiscale SEM characterization of the pristine basaltic glass substrate. Low-magnification imaging (25×, pixel size = 2 μm) reveals the overall grain morphology and size distribution of the crushed basaltic material (Figure 1a). At intermediate magnification (50×, pixel size = 1 μm), individual grain surfaces exhibit characteristically smooth, glassy textures with minimal surface roughness (Figure 1b). High-magnification imaging (100×, pixel size = 0.5 μm) provides a detailed visualization of surface features, including natural cavities, vesicular structures, and conchoidal fracture patterns typical of rapidly quenched volcanic glass (Figure 1c). These surface irregularities, while present, show no evidence of pre-existing secondary mineral phases or weathering products. The absence of surface alteration products confirms the suitability of this material as a pristine substrate for investigating the CO_2_-induced mineralization processes. Energy-dispersive X-ray spectroscopy (EDS) analysis of the unreacted grain surfaces (Figure 1d) confirms the presence of major constituent elements, consistent with the bulk XRF composition presented in Table 1. The EDS spectrum exhibits characteristic peaks corresponding to the tholeiitic basaltic composition, with prominent signals for Mg, Ca, and Fe, indicating the availability of reactive divalent cations essential for carbonate mineral precipitation.

The geochemical composition of the Stapafell basaltic glass is particularly well-suited for carbon mineralization. With a combined divalent cation oxide content (CaO + MgO + Fe_2_O_3_ = 23.8 wt %) approaching the 25 wt % threshold of highly reactive basalts, this material exhibits optimal reactivity characteristics. The elevated CaO content (11.44 wt %) is particularly important, as calcium represents the dominant cation for carbonate precipitation in CO_2_ mineralization processes. Additionally, the substantial MgO content (10.08 wt %) enhances the overall reactivity by supplying essential metallic divalent cations that facilitate rapid carbonate formation.

The amorphous nature of this basaltic glass confers substantial kinetic advantages over crystalline basaltic rocks, with dissolution rates markedly exceeding those of hydrothermally altered basalts.^63^ This enhanced reactivity results from two key factors: the metastable glassy matrix, which facilitates rapid initial dissolution, and the absence of passivating secondary mineral coatings that commonly form protective barriers in altered rocks.

### 4.2 Reactive Transport Dynamics and Carbonate Mineralization

One of the primary objectives of this study was to evaluate how advection velocity and fluid residence time influence carbonate mineral precipitation during the interaction of CO_2_-charged seawater with basaltic glass under continuous flow conditions. Two controlled flow rates, differing by an order of magnitude (0.05 mL/min and 0.005 mL/min), were employed to systematically assess the effect of residence time on mineralization kinetics and spatial precipitation patterns. These corresponded to Darcy velocities of 5.4 × 10^–6^ m/s and 5.4 × 10^–7^ m/s, respectively, yielding mean fluid residence times of approximately 8.2 h and 82 h.

Initial experiments at the higher flow rate of 0.05 mL/min for 30 days revealed no visible carbonate precipitates on basaltic glass surfaces, despite the continuous injection of CO_2_-acidified seawater. This absence of macroscopic carbonate formation was consistently reproduced in independent replicate experiments, confirming that the observation was reproducible and not an experimental artifact. High-resolution SEM-EDS characterization of postexperiment substrates from all replicates showed only minor, early-stage precrystalline phases with elevated calcium (Ca) content, akin to amorphous calcium carbonate, detectable solely at high magnification (later shown in Section 4.3 and Figure 3h). These consistent findings across replicates indicate that the 0.05 mL/min flow rate (a higher advection velocity and shorter fluid-rock contact time) was insufficient for significant carbonate nucleation and growth under the tested conditions.

Consequently, the flow rate was reduced by an order of magnitude to 0.005 mL/min, increasing the residence time. This modification created more favorable thermodynamic and kinetic conditions for carbonate nucleation and growth by allowing extended fluid-rock interaction, progressive accumulation of dissolved divalent cations, and sustained supersaturation with respect to carbonate minerals. Under these conditions, visible carbonate precipitation was successfully achieved, enabling a detailed characterization of mineralization processes and spatial distribution patterns. The observed patchy, nonuniform distribution of carbonate precipitates reflects nucleation-limited kinetics modulated by local substrate site preferences rather than fully deterministic patterns driven solely by concentration gradients.

Figure 2 presents the reactive transport experiment conducted at 0.005 mL/min. The column reactor configuration, consisting of an initial 4 cm section packed with crushed calcium carbonate followed by 36 cm of basaltic glass, is illustrated schematically alongside a photograph of the experimental setup (Figure 2a). The calcium carbonate section served as a rapid source of dissolved Ca^2+^ ions upon contact with acidified seawater, while the basaltic glass section provided the primary reactive substrate for mineral precipitation.

**Figure 2:**
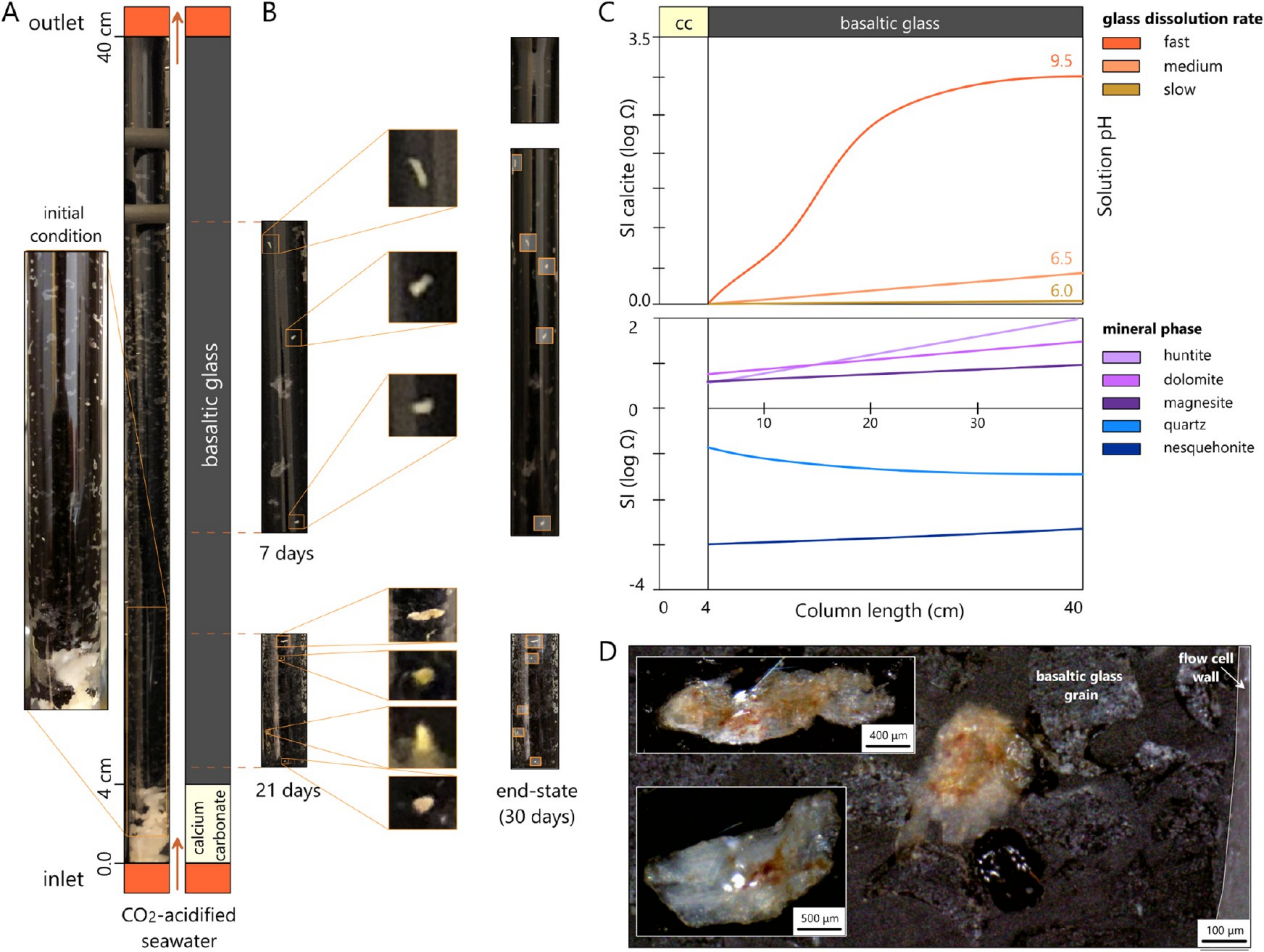
Reactive transport experiment results and geochemical modeling of CO_2_ mineralization in basaltic glass columns. (a) Experimental setup showing the column reactor configuration with an initial 4 cm calcite section and 36 cm basaltic glass section during CO_2_-acidified seawater injection at 0.005 mL/min. (b) Temporal evolution of carbonate mineral precipitation at 7, 21, and 30 days, demonstrating the spatial distribution of white carbonate accumulations along the basalt section, with preferential formation of larger precipitation patches in the latter half of the column. (c) PHREEQC v3 reactive transport simulation results: (top) calcite saturation index evolution for three basalt glass dissolution rate scenarios—fast (*k* = 1.0 × 10^–8^ mol·m^–2^·s^–1^), medium (*k* = 1.0 × 10^–9^ mol·m^–2^·s^–1^), and slow (*k* = 1.0 × 10^–10^ mol·m^–2^·s^–1^) with estimated outlet pH constraining actual rates near the medium case. (bottom) Saturation indices of carbonate minerals along the column length showing supersaturation with respect to dolomite (CaMg­(CO_3_)_2_), magnesite (MgCO_3_), and huntite (Mg_3_Ca­(CO_3_)_4_) using the medium dissolution rate (*k* = 1.0 × 10^–9^ mol·m^–2^·s^–1^). (d) High-resolution images of isolated carbonate precipitate bodies on a dark basaltic glass substrate, with scale bars indicating millimeter-scale accumulations, demonstrating the nucleation-controlled, probabilistic nature of mineralization rather than uniform growth-dominated precipitation.

Temporal monitoring at 7 and 21 days, along with end-state observations at 30 days, revealed surprising patterns of carbonate mineral accumulation (Figure 2b). White crystalline patches, identified as carbonate precipitates, appeared distributed along the entire length of the basalt-packed section. Contrary to expectations from deterministic reactive transport models, which predicted maximum precipitation near the calcite–basalt transition zone, where Ca^2+^ concentrations would be highest, a number of larger accumulations occurred in the second half of the column. This counterintuitive spatial distribution suggests that factors beyond simple concentration gradients govern precipitation patterns in the performed experiments.

The progressive pH increase along the column, from inlet pH 5.4–5.5 through calcite buffering to outlet pH 6.58–7.25, may impact precipitation patterns through pH-dependent carbonate speciation and nucleation kinetics. At lower pH, carbonate exists predominantly as H_2_CO_3_ and ${\mathrm{HCO}}_{3}^{-}$ rather than ${\mathrm{CO}}_{3}^{2-}$ , which is required for carbonate precipitation, while H^+^ ions kinetically inhibit nucleation. Higher pH increases ${\mathrm{CO}}_{3}^{2-}$ availability and reduces nucleation energy barriers, potentially accelerating nucleation rates. This can explain enhanced precipitation in later column sections despite lower Ca^2+^ concentrations: pH-controlled nucleation kinetics dominates over concentration-driven thermodynamics, though stochastic nucleation dynamics prevent deterministic spatial prediction.

The observed spatial distribution additionally reflects probabilistic nucleation dynamics.^47,64,65^ Once initial carbonate precipitation occurs, subsequent nucleation may be significantly enhanced on these newly formed carbonate surfaces—a phenomenon known as precipitation on a secondary substrate—where carbonate–carbonate interfacial energies are lower than those of carbonate–silicate interfaces. This mechanism could also contribute to the isolated, growing accumulations at initially stochastic nucleation sites rather than uniformly distributed precipitation.

High-resolution imaging of selected precipitate bodies revealed their morphologies and size distributions (Figure 2d). Individual carbonate accumulations ranged from submillimeter to several millimeters in characteristic dimensions, forming as discrete, isolated pockets rather than continuous coatings or uniformly dispersed aggregates. The contrast between white carbonate minerals and the dark basaltic glass substrate facilitated visual identification and quantification of precipitation zones. Notably, large precipitate bodies were spatially separated, with extensive regions of the column showing minimal visible mineralization, the dissolution of basaltic glass, and a continuous Ca^2+^ supply.

These observations collectively indicate that carbonate precipitation under the experimental conditions is predominantly controlled by nucleation kinetics rather than by crystal growth rates. Once a stable nucleus forms on the basaltic glass surface, subsequent growth proceeds readily due to the continuous supply of supersaturated fluid. However, nucleation events themselves are probabilistic phenomena, occurring stochastically along the column rather than in predictable locations solely on the basis of supersaturation gradients. This nucleation-limited behavior has significant implications for predicting mineralization efficiency and spatial distribution in field-scale CO_2_ storage operations, as it introduces inherent uncertainty that deterministic models alone cannot capture.

The acidity of the injected CO_2_-charged seawater was routinely monitored at the inlet and maintained at pH 5.4–5.5 throughout the 30-day experimental period. Upon entering the column, this acidified fluid first contacted the crushed calcium carbonate section, where rapid buffering occurred. Aqueous geochemical calculations using PHREEQC v3 indicate that equilibration with calcite raised the pH to approximately 6.0, accompanied by substantial dissolution of CaCO_3_ and a corresponding increase in dissolved Ca^2+^ and carbonate species concentrations. This prebuffered fluid then entered the basaltic glass section, where further buffering occurred through basaltic glass dissolution, releasing additional divalent cations (Ca^2+^, Mg^2+^, Fe^2+^) and progressively increasing the pH.

The measured pH of the outlet effluent declined systematically over the course of the experiments, from 7.25 on day 1 to 6.58 on day 30. This temporal pH evolution can be attributed to two competing processes: (1) decreasing basalt dissolution rates as reactive surfaces become passivated by secondary mineral coatings or as the most reactive glass components are preferentially leached, and (2) progressive precipitation of carbonate minerals within the column, which consumes alkalinity and shifts the carbonate equilibrium. Process (1) involves passivation by noncarbonate secondary phases, such as clay coatings on reactive basalt surfaces and suppressing further dissolution, whereas process (2) specifically refers to the precipitation of carbonate minerals that directly consume ${\mathrm{CO}}_{3}^{2-}$ and ${\mathrm{HCO}}_{3}^{-}$ , thereby reducing solution alkalinity. The observed pH decline is consistent with the system transitioning from an initial disequilibrium toward a quasi-steady state, where dissolution and precipitation rates approach balance.

Continuous differential pressure monitoring revealed no measurable change in column permeability or the pressure gradient throughout the experiments. This is consistent with the limited extent and highly localized distribution of carbonate precipitates, which did not form continuous, flow-obstructing features on the column scale.

One-dimensional reactive transport simulations were conducted to evaluate fluid geochemical evolution along the column and to constrain basalt dissolution rates (Figure 2c). Due to uncertainties in both reactive surface area and intrinsic dissolution rate constants for the basaltic glass, three scenarios spanning 2 orders of magnitude in glass dissolution rate were simulated: fast (*k* = 1.0 × 10^–8^ mol·m^–2^·s^–1^), medium (*k* = 1.0 × 10^–9^ mol·m^–2^·s^–1^), and slow (*k* = 1.0 × 10^–10^ mol·m^–2^·s^–1^) (Figure 2c, top panel).

The simulations reveal that calcite supersaturation at the column outlet varies dramatically depending on the assumed dissolution rate, ranging from near-equilibrium conditions (saturation index ∼ 0) in the slow dissolution scenario to extreme supersaturation (saturation index > 3, corresponding to >1000× supersaturation) in the fast dissolution case. Comparison of simulated outlet pH values (6.0, 6.5, and 9.5 for fast, medium, and slow dissolution scenarios, respectively) with the experimentally measured range (6.58–7.25) suggests that the actual basalt dissolution rate is comparable to or slightly higher than the medium case (*k* ≈ 1.0 × 10^–9^ mol·m^–2^·s^–1^). This rate estimate is consistent with values extrapolated from the temperature-dependent dissolution kinetics of basaltic glass across a broad pH range reported by Refs 53,59 .

Thermodynamic speciation calculations indicate that multiple carbonate mineral phases are supersaturated along the column (Figure 2c, bottom panel). Anhydrous magnesian carbonates, including dolomite (CaMg­(CO_3_)_2_), magnesite (MgCO_3_), and huntite (Mg_3_Ca­(CO_3_)_4_), are all thermodynamically stable and supersaturated, whereas hydrous magnesium carbonate phases, such as nesquehonite (MgCO_3_·3H_2_O), remain undersaturated under the experimental conditions. Calcite (CaCO_3_) exhibits the highest supersaturation and fastest precipitation kinetics, consistent with its identification as the dominant precipitate phase in postexperiment characterization.

The observed spatial heterogeneity of carbonate precipitates, manifested as discrete, isolated accumulations rather than uniform coatings (Figure 2), provides evidence for nucleation-controlled, probabilistic mineralization. Despite sustained supersaturation along the column, precipitation is patchy and distributed at various locations, including larger accumulations in the latter half, where Ca^2+^ concentrations are lower due to prior consumption. This counterintuitive distribution indicates that precipitation is primarily governed by nucleation barriers rather than by thermodynamic driving forces or simple concentration gradients alone.

Surface heterogeneity of the basaltic glass substrate, such as vesicles, valleys, curvature variations, and local differences in surface energy, undoubtedly contributes to localized crystal nucleation and growth by creating preferential nucleation sites (reduced interfacial energy barriers).^47,64–67^ These features are widely and irregularly distributed throughout the column, introducing an intrinsic source of stochasticity that aligns with and amplifies the probabilistic nature of nucleation events. Rather than imposing deterministic spatial control, this heterogeneity results in a stochastic distribution of precipitation sites, consistent with our observations of isolated precipitates separated by extensive unmineralized regions. Thus, surface-controlled effects complement and reinforce the nucleation-limited stochastic process.

Key observations supporting nucleation barriers over supersaturation dominance include: (i) no macroscopic precipitation at a higher flow rate (0.05 mL/min) despite local supersaturation potential, (ii) patchy, nonuniform patterns at a lower flow rate (0.005 mL/min) despite progressive supersaturation increase, and (iii) discrete crystal patches with no continuous coatings even in high-supersaturation zones. These features collectively point to a kinetically controlled regime in which overcoming energy barriers is rate-limiting rather than crystal growth once nuclei form (i.e., thermodynamic driving forces govern precipitation rates and spatial patterns). This indicates that, in some regions within subsurface systems, enhancing nucleation site density through favorable substrate properties, seeding with carbonate particles (secondary substrate availability), or controlled pH/alkalinity cycling may be more effective than simply increasing supersaturation levels for accelerating mineralization rates.

### 4.3 Pore-Scale Mineralization and Secondary Phase Formation

Postexperiment characterization of basaltic glass substrates revealed complex mineralization patterns and secondary phase assemblages that deviate from ideal thermodynamic-geochemical predictions, highlighting the critical role of kinetic limitations, spatial heterogeneity, and competing reactions in controlling carbon mineralization efficiency. Detailed pore-scale analysis identified four principal phenomena that collectively governed reactive transport and mineral precipitation under flow-through conditions: (1) smectite clay formation, suggested in an earlier paper to potentially prevent nucleation;^37^ (2) preferential carbonate precipitation in peripheral regions of the flow cell; (3) early-stage calcium-enriched surface phases; and (4) enhanced mineralization within cavities and vesicular features of the basaltic substrate (Figure 3). Dissolution textures (etch pits and surface roughening) that may form potential nucleation sites, as well as localized precipitation on basalt grain surfaces, are directly visualized in high-resolution SEM micrographs, which provide detailed evidence of reaction features at multiple scales.

**Figure 3:**
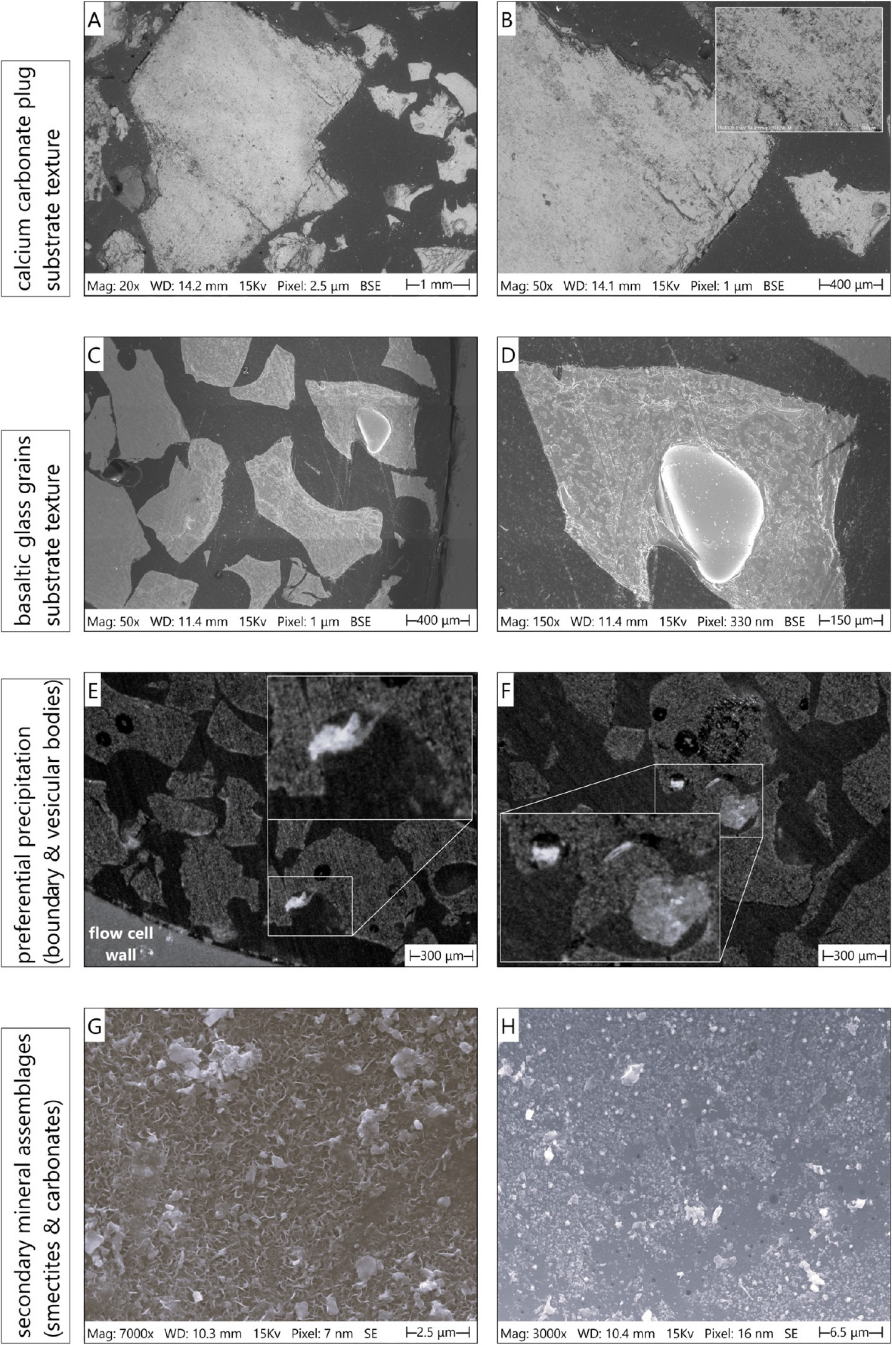
Pore-scale characterization of postexperiment substrates revealed dissolution features, spatial precipitation patterns, and secondary mineral assemblages. (a, b) Surface texture of calcium carbonate plug grains after reactive flow, showing dissolution features and surface roughening from acidified seawater interaction. (c, d) Basaltic glass grain surfaces exhibiting characteristic dissolution morphology with enhanced surface roughness. (e) Preferential carbonate precipitation in peripheral regions adjacent to the flow cell wall, attributed to reduced advection velocity in the boundary layer and enhanced fluid retention on water-wet glass surfaces. (f) Enhanced carbonate mineralization within natural cavities and vesicular features of basaltic glass grains. (g) SEM micrograph showing pervasive smectite clay formation with characteristic texture on basaltic glass surfaces. (h) Early-stage carbonate formation, characterized by crystallites and amorphous calcium-rich precipitates, on basaltic glass surfaces. The subfigures presented here do not depict location-specific features tied to a particular position in the column. The observed dissolution textures, precipitation patterns, and secondary mineral assemblages were distributed throughout the basalt section rather than confined to discrete zones. Sampling locations (distance from inlet) are indicated for reference: (a,b) 4 cm (near inlet, calcium carbonate plug region); (c,d) 22 cm (midway in the basalt section); (e) 30 cm; (f) 16 cm; (g) 10 cm; and (h) 8 cm.

#### 4.3.1 Substrate Dissolution and Surface Modification

SEM examination of postexperiment substrates revealed distinct dissolution-induced surface modifications on both calcium carbonate plug grains and basaltic glass particles (Figure 3a–d). The calcium carbonate grains, initially included in the column inlet section to provide dissolved Ca^2+^ through reaction with acidified seawater, exhibited pronounced surface roughening and the development of dissolution features, including etch pits and surface relief patterns (Figure 3a,b). These morphological changes reflect the dissolution regime imposed by the injected fluid’s acidity (pH 5.4–5.5), which released calcium ions into solution.

Basaltic glass grain surfaces similarly displayed a characteristic dissolution morphology, with significantly enhanced surface roughness compared to the pristine substrate (Figure 3c,d). Dissolution preferentially targeted structurally weak zones, creating microscale surface irregularities that subsequently acted as favorable nucleation sites for secondary mineral precipitation. The textural evolution of basaltic glass surfaces during reactive flow demonstrates the dynamic nature of fluid-rock interactions, where the simultaneous dissolution of primary phases and precipitation of secondary phases continuously modify the reactive surface area and properties available for subsequent mineralization reactions.

#### 4.3.2 Spatial Heterogeneity in Carbonate Precipitation

Detailed examination of postexperiment substrates revealed pronounced spatial heterogeneity in carbonate distribution, with three distinct localization patterns: preferential precipitation in peripheral regions near and adjacent to the flow cell wall, enhanced mineralization within cavities and vesicular features, in addition to the scattered distribution of isolated large crystals throughout the column interior (Figure 3e,f). These patterns could reflect the complex interplay between hydrodynamics, surface properties, and nucleation kinetics in controlling the spatial distribution of mineralization.

Carbonate accumulation in peripheral regions near the glass tube wall (Figure 3e) can be attributed to reduced advection velocities in the boundary layer, where viscous drag creates a low-velocity zone extending several grain diameters from the wall. This velocity reduction increases the local fluid residence time, allowing progressive accumulation of dissolved cations and sustained supersaturation with respect to carbonate minerals. Additionally, the water-wet properties of the borosilicate glass tube may promote preferential fluid retention in wall-adjacent regions, further enhancing residence time effects. The hydrophilic glass surface facilitates the formation of a thin aqueous film that experiences minimal advective transport, thereby creating conditions conducive to diffusion-controlled mineralization.

The vesicular and cavity-rich nature of basaltic glass grains provides preferential nucleation sites for carbonate precipitation (Figure 3f). Surface irregularities, including natural cavities, vesicles, and conchoidal fracture features, offer geometrically favorable sites for heterogeneous nucleation by reducing the critical nucleus size through decreased interfacial energy. Within these microenvironments, fluid stagnation zones develop where advective transport is minimal, allowing diffusion-dominated conditions that favor progressive supersaturation buildup.

Detailed examination of postexperiment substrates revealed pronounced spatial heterogeneity in carbonate distribution, with preferential precipitation in peripheral regions near the flow cell wall, enhanced mineralization within natural cavities and vesicular features, and critically isolated large accumulations throughout the column interior. These patterns reflect the complex interplay among local hydrodynamics, surface properties, and nucleation kinetics.

Surface heterogeneities of basaltic glass may create geometrically and energetically favorable sites for heterogeneous nucleation by reducing interfacial energy barriers and promoting fluid stagnation in sheltered regions. These features are widely and irregularly distributed across grains throughout the column, providing a heterogeneous but intrinsically stochastic template of potential nucleation sites. Rather than driving deterministic precipitation patterns, this widespread heterogeneity contributes to the observed probabilistic, patchy distribution: only a small fraction of apparently favorable sites progress from initial Ca enrichment to visible crystal formation, while extensive regions remain unmineralized despite supersaturation and surface exposure.

This pore-scale selectivity is observed at the column scale and supported by (i) elevated Ca concentrations across surfaces throughout the column without corresponding uniform carbonate growth, indicating that thermodynamic favorability alone is insufficient; (ii) isolated, discrete precipitates separated by large unreacted areas, inconsistent with purely surface-templated or growth-dominated processes; and (iii) co-occurrence of cavity-enhanced precipitation within interior patches, suggesting probabilistic site activation modulated by local surface properties rather than strict morphological control. Together, these observations directly link pore-scale processes to the column-scale probabilistic distribution, reinforcing the mechanisms discussed: that nucleation barriers, amplified by distributed heterogeneities, are the primary rate-limiting step.

#### 4.3.3 Secondary Mineral Assemblages: Carbonates and Clays

The dissolution of basaltic glass released substantial quantities of divalent cations (Ca^2+^, Mg^2+^, Fe^2+^) and silica into the solution, creating conditions for both carbonate and phyllosilicate precipitation. Postreaction SEM characterization revealed two distinct secondary mineral assemblages: clay minerals exhibiting characteristic sheet-like morphology consistent with smectite group phyllosilicates (Figure 3g), and early-stage calcium-enriched phases representing incipient carbonate formation (Figure 3h). The smectite phases displayed a honeycomb-like texture typical of dioctahedral smectites, forming patchy (nonuniform, discontinuous) coatings on basaltic glass surfaces. The presence of smectite may create activation energy barriers that kinetically inhibit carbonate nucleation on coated surfaces, analogous to the smectite-induced inhibition previously suggested for basaltic systems.^37^ The formation of smectite clays (Figure 3g) represents an additional competing kinetic factor.^37,68–70^ Patchy clay coatings may further increase local nucleation barriers on affected surfaces, contributing to the limited spatial extent of carbonate mineralization despite favorable conditions elsewhere. This interplay between stochastic nucleation on heterogeneous surfaces and secondary phase passivation underscores the kinetic dominance over thermodynamic predictions for controlling pore-scale carbon mineralization.

The formation of smectite clays under our experimental conditions (80 °C, pH 6.58–7.25) is consistent with previous studies of basalt alteration in CO_2_-charged aqueous systems.^37^ Thermodynamic calculations indicate that smectite stability is favored at circumneutral pH and temperatures below 100 °C, conditions where the Mg^2+^/H^+^ activity ratio promotes phyllosilicate formation over carbonate precipitation. The pervasive nature of smectite coatings on basaltic glass surfaces suggests that clay formation represents a competing sink for divalent cations that would otherwise contribute to carbonate mineralization.

The inferred formation of smectite-like clays was based on their characteristic sheet-like morphology and honeycomb texture observed at magnifications >1000× in SEM images; however, without detailed crystallographic confirmation (e.g., XRD or TEM-SAED), we cannot exclude the presence of amorphous phases or mineral mixtures, particularly under high-ionic-strength seawater conditions. Mineral phase identification and quantification via XRD analysis of representative samples showed no detectable new peaks, consistent with the small fraction of surface phases relative to the bulk glass substrate, which limits detection sensitivity in the XRD volume-averaged approach.

In contrast to the predicted formation of magnesian and ferroan carbonates (magnesite, siderite, and ankerite) suggested by equilibrium thermodynamic modeling, the experimentally observed carbonate phases were predominantly calcium rich. High-resolution EDS mapping revealed elevated calcium concentrations across extensive surface areas (Figure 3h), indicating widespread nucleation of calcium carbonate precursors, though only a limited number of these nucleation sites developed into macroscopic crystalline precipitates. This observation suggests that, while thermodynamic driving forces for carbonate precipitation exist throughout the system, kinetic barriers to rapid growth limit the spatial extent of actual mineralization.

Three mechanistic explanations may account for the absence of Mg–Fe–Ca carbonates, despite their thermodynamic stability and supersaturation in the reacted fluids: (1) preferential sequestration of Mg^2+^ and Fe^2+^ by smectite formation, depleting solution concentrations below the threshold required for mixed carbonate nucleation; (2) kinetic inhibition of carbonate nucleation due to surface passivation by clay (smectite) coatings, which create activation energy barriers for heterogeneous nucleation; and (3) slow-moving crystal growth kinetics resulting from unfavorable aqueous Me^2+^/ ${\mathrm{CO}}_{3}^{2-}$ activity ratios that extend nucleation induction times beyond experimental time scales. This has collective implications for reactive transport modeling. Kinetic frameworks based solely on transition state theory (TST)-derived rate laws, which do not explicitly account for nucleation barriers or the influence of aqueous activity ratios on precipitation kinetics, may overestimate carbonatization rates by several orders of magnitude.^37^ Accurate prediction of CO_2_ mineralization efficiency in basaltic systems requires incorporation of nucleation-based kinetic models that capture the stochastic nature of carbonate formation as well as the effect of solution stoichiometry.^71^

The preferential formation of calcium carbonate over magnesium phases reflects differences in precipitation kinetics.^37^ Calcite exhibits faster nucleation rates and lower activation energies compared to magnesite, particularly at temperatures below 100 °C.^72^ The higher hydration energy of Mg^2+^ relative to Ca^2+^ imposes a substantial kinetic barrier to magnesite precipitation, requiring either elevated temperatures (>150 °C) or extended reaction times to achieve significant magnesian carbonate formation. These kinetic limitations are exacerbated in flow-through systems, where residence times are constrained, further favoring rapid calcite precipitation over slower-forming magnesian phases.

### 4.4 Pore Space Characterization of Vesicular Basalts

Three distinct facies of Icelandic basalts, representing the spectrum of vesicularity expected in basaltic reservoirs, were characterized using integrated multiscale imaging, including visible light photography, high-resolution X-ray microcomputed tomography (micro-CT), and scanning electron microscopy (SEM) (Figure 4). These samples encompass: (1) dense flow-interior facies (Sample A) with limited vesicle connectivity, (2) transitional facies (Sample B) with intermediate connectivity, and (3) highly vesicular flow-top facies (Sample C) with well-developed pore networks. This suite provides systematic insight into how vesicle connectivity and pore network topology control the reactive transport behavior in basaltic CO_2_ storage reservoirs.

**Figure 4:**
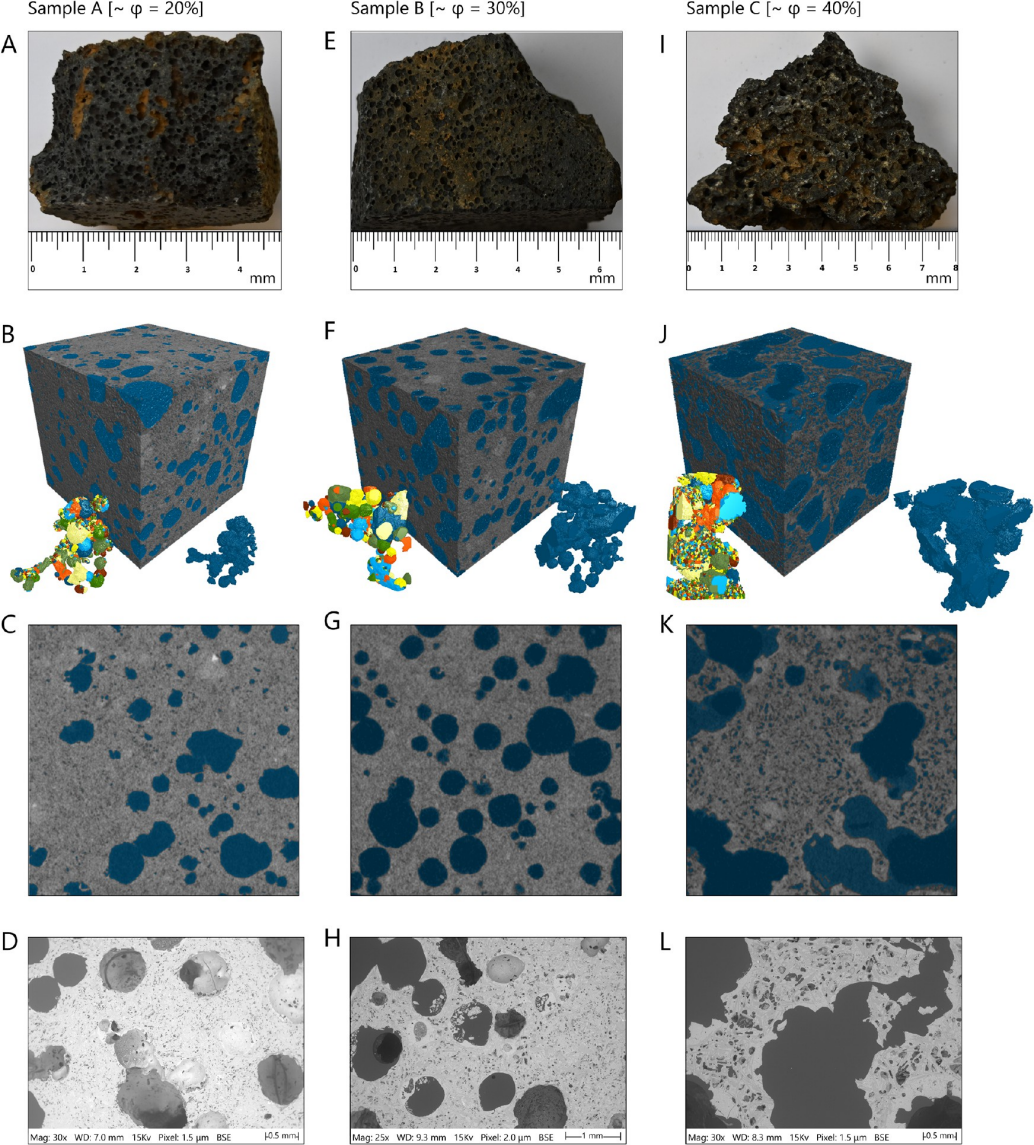
Multiscale characterization of three Icelandic basalt facies representing the spectrum of vesicularity in basaltic reservoirs. Columns from left to right: Sample A (dense flow-interior facies) (A–D), Sample B (transitional facies) (E–H), and Sample C (highly vesicular flow-top facies) (I–L). First row: Visible light photography shows macroscale textural features and vesicle distributions. Second row: Three-dimensional micro-CT reconstructions with segmented pore space (blue), the largest connected-component pore network, and the corresponding extracted individual pore bodies derived from PNM. Third row: Vertical profiles through segmented volumes illustrate the spatial distribution and connectivity of vesicular networks. Fourth row: High-resolution SEM micrographs characterize vesicle wall thickness, intervesicle connectivity, and matrix microporosity. Note the progressively increasing matrix microporosity from Sample A through C, which contributes to overall network connectivity beyond the macrovesicular structure alone and helps explain the expected superior hydraulic conductivity of Sample C relative to Sample B.

Macroscale photographic analysis reveals distinct textural characteristics across the three facies (Figure 4, first row). Sample C displays abundant vesicles ranging from millimeter to centimeter scale, with elongated geometries reflecting flow-induced deformation during magma emplacement. Sample B exhibits moderate vesicularity with a bimodal size distribution, comprising small (1–2 mm) spherical vesicles and larger (>5 mm) irregular voids. Sample A appears substantially denser and more massive, with scattered, isolated vesicles discernible only upon close examination of macroscale features throughout the specimen.

Three-dimensional micro-CT reconstructions (Figure 4, second row), coupled with vertical profiles through the segmented volumes (Figure 4, third row), quantify fundamental topological differences between the basaltic facies. For each sample, two specimens were imaged and analyzed to capture the inherent heterogeneities. Segmented porosity analysis reveals total porosities of 18.3–21.0% (±2%) for Sample A, 30.5–31.3% (±3%) for Sample B, and 41.4–41.5% (±5%) for Sample C. Region-of-interest (ROI) size-dependent variations in measured porosity were observed, particularly for Sample C, where the inclusion of large coalesced vesicular bodies introduced notable pore volume increases, resulting in 1–5% variability across the sample suite based on the selected ROI size.

The fraction of porosity contributing to the largest connected-component pore network (visualized alongside the 3D segmented volumes in the second row) represents a critical parameter for controlling bulk permeability. It differs dramatically across samples: only 1.3–3.8% distributed over multiple disconnected ROIs in Sample A, 12.1–13.3% in Sample B, and 31.7–32.2% in Sample C. This contrast indicates that Sample A contains predominantly isolated, noneffective porosity, while Sample C exhibits nearly complete pore connectivity throughout the vesicle network. The spatial distribution and connectivity patterns are clearly visualized in the vertical profiles presented in the third row, where Sample C shows several continuous, connected pathways; Sample B reveals partially connected networks with interruptions; and Sample A displays smaller, predominantly isolated pore clusters.

High-resolution SEM imaging provides micrometer-scale characterization of vesicle morphology, surface texture, and intervesicle connectivity (Figure 4, fourth row). In Sample C, vesicle walls are thin and frequently breached, creating direct hydraulic connections between adjacent voids through intervesicle windows ranging from tens of micrometers to millimeters in diameter. These breached connections enable efficient fluid transport throughout the pore network. Sample A reveals predominantly isolated vesicles with thick, intact walls and rare intervesicle connections. Surface texture analysis reveals significant matrix microporosity in Sample C, which progressively decreases in size and abundance from Sample C to Sample A, reflecting variations in cooling rate and degassing efficiency during lava emplacement.

Quantitative pore structure analysis reveals systematic trends that correlate with bulk porosity. Mean pore diameter increases progressively: 0.25–1.4 mm in Sample A, 0.6–1.5 mm in Sample B, and 0.7–2.2 mm in Sample C. Sample C exhibits elongated, interconnected vesicular structures forming extensive void spaces spanning several millimeters without narrow constrictions, whereas Sample A contains smaller, more isolated vesicular bodies. Measured pore throat diameters—the critical bottlenecks controlling permeability—range from 0.15–0.40 mm in Sample A, 0.25–0.75 mm in Sample B, and 0.3–1.0 mm in Sample C. The narrow throats in Sample A, combined with poor connectivity, yield lower expected fluid permeability, while the wider, well-connected throats in Sample C enable highly permeable percolation pathways.

Pore coordination number distributions derived from the 3D micro-CT data sets reveal striking differences in the topological architecture of the pore network. Sample C exhibits coordination numbers ranging from 1 to 4, with a modal value of 2, indicating that most vesicles connect to two neighboring voids, creating predominantly serial or chain-like connectivity. Sample B shows similar coordination number distributions but with reduced overall connectivity due to lower vesicle abundance and incomplete coalescence. Sample A displays extremely low coordination, with the majority of vesicles either completely isolated (coordination number = 0) or connected to only a single neighbor (coordination number = 1), confirming the absence of percolating flow networks capable of sustaining effective fluid transport.

The integrated multiscale characterization demonstrates the systematic evolution of pore network architecture from dense flow interiors through transitional zones to highly vesicular flow tops. This progression reflects the interplay between vesicle nucleation density, bubble growth and coalescence during magma ascent and lateral flow, and the cooling rate during solidification. The dramatic differences in connected porosity fraction (spanning from <4% to >30%), despite more modest variations in total porosity (18–41%), underscore the critical importance of pore connectivity topology rather than simple porosity magnitude in controlling hydraulic properties and suitability for CO_2_ storage applications.

Quantitative pore network modeling (PNM) of the segmented micro-CT volumes provides a detailed statistical characterization of individual pore bodies (vertices) and connecting throats (edges) across the three basalt facies (Table 2). PNM analysis extracts topological and geometric properties from the three-dimensional pore space, enabling systematic comparison of pore and throat size distributions, connectivity metrics, and network architecture parameters that control fluid transport.

Pore body and throat properties exhibit broad, highly skewed distributions (high standard deviations often exceeding means, e.g., volume in Sample B, 0.16 ± 0.48 mm^2^), indicative of the multiscale, multilevel pore heterogeneity typical of vesicular basalts. This arises from primary vesiculation producing vesicles across orders of magnitude in size, combined with potential secondary processes that further broaden the distribution toward heavy-tailed behavior. Consequently, arithmetic means are reported for consistency with prior studies, but the 5th–95th percentiles are emphasized as more representative of central tendencies in these non-Gaussian data sets.

Vertex (pore body) analysis reveals systematic variations in pore geometry and connectivity across the three facies. Sample B exhibits the largest mean pore volumes (0.16 ± 0.48 mm^2^) and surface areas (0.74 ± 1.52 mm^2^), with equivalent diameters reaching 0.35 ± 0.33 mm and 95th percentile values approaching 1 mm. These substantial pore dimensions reflect the transitional nature of this facies, where moderate vesicularity combines with partial coalescence to create intermediate-scale void spaces. Sample C, despite higher total porosity (41%), displays smaller mean pore volumes (0.06 ± 0.12 mm^2^) due to the significant share of porosity identified in the basalt texture itself, and equivalent diameters (0.14 ± 0.10 mm), indicating that the elevated porosity derives from abundant small-to-intermediate pore bodies (in the basalt framework and as vesicles) besides the many large coalesced voids. Sample A exhibits the smallest pore volumes (0.01 ± 0.04 mm^2^) and equivalent diameters (0.15 ± 0.09 mm), consistent with its dense, poorly vesicular character.

The connectivity metric, which represents the number of throats connected to each pore body, provides critical insights into the network topology. Sample C exhibits the highest mean connectivity (5.6 ± 5.1), with 95th percentile values reaching 12.5, indicating a well-developed pore network where individual vesicles connect to multiple neighbors. Sample B displays intermediate connectivity (3.2 ± 2.6), reflecting partial network development. Sample A exhibits a connectivity value of 3.2 ± 2.1, which paradoxically appears comparable to Sample B despite dramatically lower bulk connected porosity (1.3–3.8% versus 12.1–13.3%). This apparent contradiction reflects the nature of PNM analysis in poorly connected systems: the connectivity values represent local coordination within small, isolated pore clusters rather than network-scale connectivity. Sample A comprises numerous isolated vesicle groups, each internally showing modest connectivity, but with minimal intercluster connections preventing the formation of percolating pathways. The large standard deviations across all samples (approaching or exceeding mean values) underscore the pronounced heterogeneity characteristic of vesicular pore networks.

Edge (throat) analysis quantifies the critical bottlenecks controlling permeability. Sample B exhibits substantially larger mean throat diameters (0.21 ± 0.24 mm) compared to Samples C and A (both 0.08 ± 0.06–0.07 mm), with 95th percentile values reaching 0.72 mm. These wide throats, combined with the intermediate total porosity and connectivity, position Sample B as having favorable hydraulic properties despite not exhibiting the highest vesicularity. The throat lengths in Sample B (mean 0.64 ± 0.43 mm, 95th percentile 1.49 mm) exceed those in Samples C (0.28 ± 0.21 mm) and A (0.24 ± 0.13 mm), reflecting the elongated, tubular geometry of intervesicle connections in the transitional facies. Throat cross-sectional areas follow similar trends, with Sample B (0.09 ± 0.23 mm^2^) having larger values than Samples C and A (0.02 ± 0.04 mm^2^ and 0.01 ± 0.02 mm^2^, respectively).

Pore-specific surface area (SSA_pore_), decreases systematically from 9.0 mm^–1^ (dense flow-interior facies, Sample A) to 4.6 mm^–1^ (transitional facies, Sample B) and 2.8 mm^–1^ (highly vesicular flow-top facies, Sample C), reflecting a progressive increase in mean pore size and vesicle interconnectivity across the vesicularity spectrum (mean porosity from 19.5% to 41.5%). This inverse relationship indicates a petrophysical trend in basaltic rocks, where higher vesicularity favors larger, aperture-dominated pore networks, which, in turn, diminish the surface-to-volume ratio. Bulk SSA (SSA_bulk_ = SSA_pore_ × ϕ), normalized to rock volume, follows suit at approximately 1.8 → 1.4 → 1.2 mm^–1^.

The combination of pore and throat metrics explains the observed permeability hierarchy. Sample C achieves high permeability through abundant, well-connected small-to-intermediate pores, despite modest individual throat diameters—the sheer number of parallel pathways compensates for individual constriction sizes. Sample B maintains intermediate-to-high permeability through fewer but substantially wider throats connecting larger pore bodies, with a different architectural solution achieving a similar hydraulic conductivity. Sample A exhibits far lower permeability due to the combination of small pore volumes, narrow throats, and critically poor network-scale connectivity, despite local clustering.

### 4.5 Environmental Implications for Reactive Flow Engineering in Vesicular Basalt Reservoirs

#### 4.5.1 Hydrodynamic Control on Precipitation Dynamics

The observed strong dependence of carbonate precipitation on flow rate (and thus hydrodynamic residence time) underscores the fundamental competition between advective solute transport and nucleation/growth kinetics in controlling precipitation dynamics. At higher flow rates (0.05 mL/min and shorter residence times), local supersaturation fails to reach the critical threshold required to overcome nucleation energy barriers at most surface sites, resulting in minimal macroscopic carbonate precipitation. Conversely, at lower flow rates (0.005 mL/min, longer residence times), extended fluid-rock contact in low-velocity zones allows supersaturation to build sufficiently, enabling probabilistic nucleation followed by mineral growth, manifested as the patchy, heterogeneous patterns observed experimentally.

This advection–nucleation balance can be quantitatively described through the dimensionless Damköhler number (Da),^73–75^ commonly defined as the ratio of a characteristic transport time scale to a characteristic reaction time scale: Da = τ_transport_/τ_reaction_, or equivalently Da ∝ reaction rate/transport rate (with transport here referring to advection in flow-through systems). High Da values favor nucleation-dominated regimes, in which precipitation preferentially occurs in diffusion-limited, low-velocity microenvironments rather than uniformly across available surfaces, consistent with our observations of site-specific, patchy carbonate formation.

Recent pore-scale studies of barite precipitation in fractured porous media^73^ identified analogous regime transitions: surface-dominated precipitation at low Da (high flow, advection control) versus precipitation within alteration zones or sheltered sites at high Da (low flow, nucleation control), modulated further by pH effects on supersaturation and kinetics. Beyond the classical Damköhler framework, the role of nucleation kinetics in controlling precipitation onset can be understood through the characteristic time scale for nucleation, τ_nuc_ ∼ 1/*J*_nuc_, where *J*_nuc_ is the nucleation rate (nuclei per unit area per time). When this nucleation time scale is compared to the advective residence time, τ_adv_ ∼ *L*/*u* (where *L* is a characteristic length scale and *u* is flow velocity), the resulting ratio effectively determines whether sufficient nuclei can form before supersaturated fluid is advected away. At high Da (slow flow and long residence time relative to reaction), nucleation barriers can be overcome locally, enabling precipitation even at moderate supersaturation. Conversely, at low Da (fast flow and short residence time), nucleation may be kinetically suppressed despite thermodynamic supersaturation, as insufficient time exists for critical nuclei to form and grow before fluid renewal.

The spatial distribution of precipitation is thus governed by local variations in both supersaturation and hydrodynamic residence time.^47,70,73,76,77^ In flow-through systems, this manifests as preferential precipitation in low-velocity zones, where Da is locally elevated, even when bulk flow rates remain high. This mechanistic coupling between nucleation barriers and advective transport provides a physically sound framework for predicting not only *when* precipitation occurs, but *where* it preferentially localizes within heterogeneous porous media. Such quantitative frameworks enable the prediction of regime transitions in systems where both supersaturation generation and nucleation barriers are spatially heterogeneous.^78,79^

Similarly, microfluidic observations of calcium carbonate precipitation^47,79–81^ revealed that flow heterogeneity and localized supersaturation hot spots dictate precipitation patterns, with nucleation preferentially occurring at surface defects, roughness features, or low-shear zones where residence time is effectively extended. The competition between nucleation rate and advection rate thus provides a broadly transferable framework for predicting regime shifts and spatial heterogeneity in precipitation, even if quantitative thresholds (e.g., exact Da, τ_nuc_ or τ_adv_ values for regime boundaries) remain mineral- and chemistry-specific.

We note that while dimensionless numbers offer valuable predictive insight under simplifying assumptions (e.g., steady-state flow, uniform initial conditions, single-mineral systems), they may not fully capture transient effects, evolving surface reactivity, multimineral coupling, or feedback mechanisms between precipitation and permeability modification in complex natural systems.^78,82,83^ Nonetheless, these quantitative frameworks provide a rigorous foundation for interpreting our experimental observations and extrapolating hydrodynamic controls on carbonate precipitation to subsurface geological environments.

#### 4.5.2 Vesicular Pore Architecture and Precipitation-Induced Clogging

Vesicular features in basaltic rocks form during magma solidification through a cascade of processes: volatile exsolution, bubble nucleation, growth, migration, and coalescence.^84–86^ As magma ascends, the decrease in confining pressure reduces volatile solubility, driving supersaturation and triggering heterogeneous bubble nucleation on crystal surfaces or homogeneous nucleation within the melt.^87^ These nascent bubbles grow through diffusive influx of volatiles from the surrounding melt and decompression-driven expansion.^85,87^ During continued ascent and lateral flow, bubbles coalesce when interbubble films rupture, creating interconnected voids whose extent depends on ascent rate, bubble number density, and melt viscosity.^87,88^ Upon solidification, this vesiculation history is preserved as a frozen record, manifesting as vesicles ranging from submillimeter spherical pores to elongated, centimeter-scale interconnected structures that fundamentally control hydraulic properties.

A common misconception is that vesicles form only isolated, ineffective porosity. While isolated vesicles certainly exist within flow interiors and transitional zones,^86,89,90^ laboratory measurements and theoretical models demonstrate high connectivity above a percolation threshold of approximately 30% porosity.^91–93^ This critical threshold is commonly exceeded in basaltic flow tops,^86,94^ where porosities reach 40–50% or higher, as confirmed by our Sample C characterization showing 41% total porosity with 32% connected porosity.

Permeability evolution with porosity in vesicular basalts follows nonlinear relationships that are fundamentally different from those in granular sedimentary rocks, driven primarily by differences in pore network topology. Pore coordination number, which is the number of throats connecting each pore body to its neighbors, serves as the critical metric quantifying these topological differences. As illustrated in Figure 5a, reservoir-quality sandstones exhibit coordination numbers ranging from 4 to 6, reflecting the geometrically regular packing of approximately spherical grains. This high coordination creates multiple parallel flow pathways, imparting substantial redundancy, such that blockage of individual pore throats has a limited impact on bulk permeability, since fluids redistribute through alternative connections.

**Figure 5:**
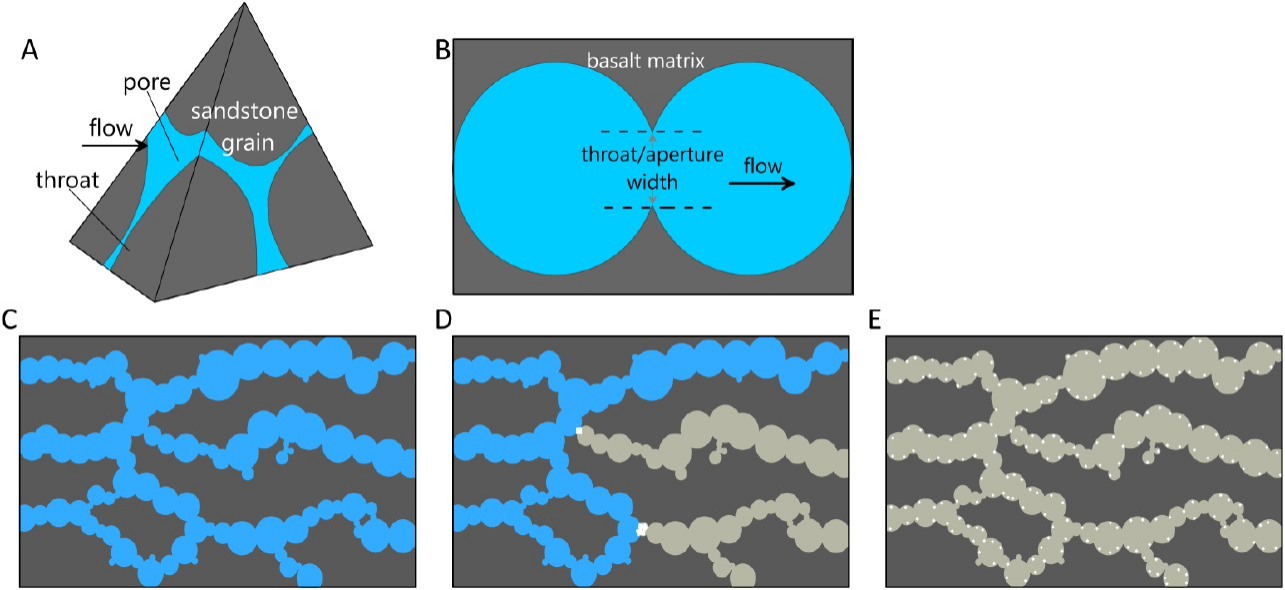
Pore network topology and precipitation-induced clogging scenarios in sandstone versus basalt reservoirs. Top row: Comparison of pore and throat architecture shows fundamental differences in network connectivity. (a) Representative sandstone pore network with a high coordination number (4–6 connections per pore), creating multiple parallel flow pathways with substantial redundancy that maintains permeability even when individual throats become blocked. (b) Vesicular basalt pore network displaying a low coordination number (predominantly 2 connections per pore), creating serial or chain-like connectivity vulnerable to marked permeability loss if critical throats are occluded. Despite larger throat diameters in basalts compared to sandstones, the low-redundancy topology renders basaltic networks more susceptible to precipitation-induced impairment. Bottom row: Precipitation scenarios and their differential impacts on permeability in basaltic pore networks. (c) Schematic representation of percolation pathways in highly vesicular basalt reconstructed from micro-CT imaging, illustrating the tenuous, predominantly serial connectivity characteristic of vesicular networks. (d) Scenario 1: Large, isolated carbonate precipitates (white masses) form within primary flow percolation pathways, representing the classical throat-clogging case that can rapidly disconnect serial pathways. (e) Scenario 2: Numerous small, distributed carbonate precipitates (white patches) preferentially form in pore bodies, as observed in our experiments, systematically narrowing multiple flow pathways, eliminating limited redundancy, and causing disproportionately severe permeability loss, even at modest precipitated volumes. Figure 5d and e illustrates two complementary end-member scenarios. Scenario (e) is more closely aligned with the patchy, low-velocity-site preference documented in our column experiments and has particularly serious implications for injectivity in vesicular basaltic reservoirs, as discussed in detail in Ref 95.

In contrast, vesicular basalts exhibit dramatically lower coordination numbers (1–3, with a modal value of 2), reflecting their fundamentally different origin through bubble coalescence rather than grain packing (Figure 5b). This creates serial or chain-like connectivity rather than three-dimensional networks, where many vesicles connect to only one or two neighbors, establishing a low-redundancy serial flow architecture. Notably, vesicular basalts often display larger pore apertures (throat diameters of 0.3–1.0 mm in Sample C) than sandstone matrix pores (typically <0.05–0.1 mm), yet their low coordination number renders them more vulnerable to permeability impairment despite these wider individual connections.

The difference in coordination number between sandstones (4–6) and basalts (1–3) fundamentally alters reactive transport vulnerability to mineral precipitation. In high-coordination sandstones, precipitation must occlude a substantial fraction of pore throats—approximately 75%, based on percolation theory—before causing a significant reduction in permeability, as fluids continuously redistribute through alternative pathways. In low-coordination basalts, however, the occlusion of even a single critical throat can sever connectivity across entire network portions, causing major local permeability loss. The permeability–porosity relationship becomes highly nonlinear with threshold behavior: modest precipitation volumes can trigger disproportionate permeability reductions once critical bottlenecks seal. For coordination-2 systems, percolation theory predicts bond percolation thresholds near 50%, meaning occlusion of approximately half the throats causes complete loss of connected pathways—substantially lower resilience than coordination-6 sandstones, requiring ∼75% blockage.

The pronounced spatial heterogeneity characteristic of vesicular basalts further exacerbates this topological vulnerability. Vesicle size distributions can span two to three orders of magnitude—from submillimeter spherical voids to centimeter-scale elongated cavities—with the smallest intervesicle connections representing critical choke points controlling bulk permeability. Preferential nucleation and growth of secondary minerals in these narrow throats, driven by locally elevated supersaturation and favorable surface properties for heterogeneous nucleation, amplify the tendency toward early permeability impairment.

The interaction between mineral precipitation and fluid flow operates as a coupled, self-reinforcing feedback system with fundamentally different dynamics compared to those in sandstone reservoirs. Under initial uniform injection conditions in unreacted basaltic samples, fluid velocity exhibits pronounced spatial heterogeneity, reflecting the irregular vesicle geometry and variable throat dimensions. High velocities concentrate in large vesicles and wide connecting throats, the primary flow highways through the network, while stagnant or slowly recirculating fluid occupies smaller vesicles and dead-end pores branching from the primary pathways. This velocity heterogeneity establishes the initial template for reactive transport partitioning.

Carbonate precipitation is initiated where local supersaturation reaches critical nucleation thresholds, which may not necessarily coincide with high-velocity flow paths. As documented in our column experiments, nucleation appears to be stochastic, dominated by surface properties, microscale heterogeneities, and local residence time, rather than simply tracking regions of highest bulk supersaturation. Once stable nuclei form, crystal growth proceeds at rates determined by the interplay of supersaturation, available growth surface area scaling with the mass of the secondary crystals, and mass transfer limitations. Critically, the growth of crystals progressively narrows pore throats, locally increasing the fluid velocity and shear stress in constricted regions.

The probabilistic nature of the precipitation location creates fundamentally different permeability evolution scenarios (Figure 5c–e). If large carbonate masses nucleate and grow within primary flow percolation pathways, then the precipitates effectively occupy and disconnect flow highways (Figure 5d). These small yet chunky precipitation volumes may cause disproportionate reductions in permeability by creating serial bottlenecks that restrict flow throughout the connected network.

Our recent pore-scale reactive transport simulations^95^ reveal a counterintuitive finding with significant implications for reservoir management: numerous small, distributed precipitates cause more severe permeability degradation than a few large, isolated accumulations (Figure 5e). While conventional intuition suggests that large carbonate patches pose the primary threat to injectivity, distributed fine-scale precipitation systematically reduces the effective diameter of multiple parallel pore throats, eliminating the limited redundancy that might otherwise preserve permeability in low-coordination networks. This distributed clogging progressively converts the pore network from a poorly connected three-dimensional structure toward isolated, disconnected vesicle clusters, catastrophically reducing permeability despite a very modest overall porosity loss. A low coordination number amplifies these precipitation-induced reductions in permeability. In coordination-2 systems, where each pore connects to only two neighbors, blockage of any single connection separates the entire linear chain, partitioning the network into isolated upstream and downstream segments with no alternative bypass pathways.

Figure 5d and e illustrates two complementary end-member precipitation scenarios. Panel (d) represents the classical throat-clogging case in primary flow highways, which can rapidly disconnect sequential pathways. Panel (e) depicts distributed pore-body clogging, where numerous small precipitates preferentially form in pore bodies, as observed in our experiments, thereby deteriorating the permeability at a faster rate. This distributed mechanism systematically narrows multiple flow percolation pathways, eliminates the limited redundancy of low-coordination networks, and causes disproportionately severe permeability loss even at modest precipitated volumes. The low coordination number (modal value: 2) amplifies this effect: blockage of a single connection severs entire network segments. Scenario (e) is therefore more closely aligned with the low-velocity-site preference and has particularly serious implications for injectivity in vesicular basaltic reservoirs, as discussed in detail in our previous work.^95^

While no detectable permeability reduction was observed in the flow-through columns—likely due to the higher coordination number of the granular packing of basaltic glass, the limited precipitate volume, and the distributed, patchy, low-velocity-site-preferred nature of precipitations—the pore-scale imaging and PNM results indicate that analogous localized precipitation in low-coordination-number vesicular basalt networks could lead to substantially greater permeability impairment, particularly when precipitates form in or bridge pore throats. This topological vulnerability is further supported by our previous reactive lattice Boltzmann simulations,^95^ which showed order-of-magnitude reductions in permeability under throat-clogging conditions, even at low precipitated volumes (under low porosity reduction). Thus, the risk of clogging in natural basaltic reservoirs remains an important consideration for field-scale CO_2_ sequestration despite the absence of macroscopic flow impairment in the present column experiments.

The clogging threshold estimates presented here are an exploratory PNM analysis in light of the presented results and previous reactive LBM simulations (e.g., Ref. 95 ), which are conditional on the specific low-coordination vesicular networks imaged and assumed precipitation scenarios. They should, therefore, be interpreted as order-of-magnitude guidance rather than precise numbers or universal predictions for all basaltic reservoirs, which exhibit wide variability in lava flow facies, alteration state, fracture dominance, and pore network topology. While the pronounced difference in coordination number (1–3 in vesicular basalts vs 4–6 in sandstones) highlights a fundamental topological vulnerability to permeability impairment from modest precipitation volumes, actual field-scale outcomes will depend on site-specific factors, including the degree of alteration, presence of fractures, and spatial distribution of precipitation. Future work incorporating larger sample sets, multiresolution imaging, and probabilistic precipitation scenarios under reactive transport modeling will be essential to refine these insights and improve predictive capability for CO_2_ mineralization in basaltic systems.

#### 4.5.3 Seawater versus Freshwater for Carbon Mineralization

The substitution of seawater for freshwater introduces geochemical constraints that alter the mineralization pathways and efficiency. Seawater’s elevated Mg^2+^ (∼53 mM) and ${\mathrm{SO}}_{4}^{2-}$ (∼28 mM) concentrations create competing reactions that systematically reduce carbon mineralization, as observed and also reported in literature.^29,35,36^

The high Mg^2+^/Ca^2+^ ratio (∼5:1) in seawater thermodynamically favors magnesian carbonates and kinetically favors aragonite.^96–101^ Aragonite tends to form elongated acicular crystals, creating fibrous precipitates that can substantially reduce permeability while minimally affecting porosity. Magnesian carbonate precipitation kinetics, however, remain prohibitively slow below 100 °C.^72,102,103^ Experimental evidence consistently demonstrates this kinetic barrier: Voigt et al.^35^ achieved magnesite formation only at elevated pCO_2_ (16 bar) after 140 days, while Rosenbauer et al.^29^ observed ferroan magnesite requiring 100 °C and months-long reaction times. During our experimental conditions, calcium carbonate (inferred as aragonite based on seawater chemistry literature) dominated over any magnesite (not observed in crystalline form), despite its appearance in thermodynamic-geochemical predictions.

Dissolved Mg^2+^ simultaneously inhibits calcite precipitation through multiple mechanisms: surface adsorption reduces growth rates by up to 90%,^104,105^ incorporation creates lattice strain,^106,107^ and competitive complexation reduces free Ca^2+^ activity.^39^ Our flow-through experiments confirmed this inhibition, requiring order-of-magnitude increases in residence time to achieve visible precipitation compared with typical freshwater systems.^56,62^

Secondary mineral competition further diminishes the mineralization efficiency. Our experiments revealed notable instances of smectite formation, corroborating observations by Voigt et al.^35^ and Gysi and Stefánsson,^108,109^ which documented Mg-saponite consuming available Mg^2+^. This phyllosilicate precipitation represents an irreversible cation sink^35,110–112^ while potentially reducing permeability through pore-throat occlusion^113–116^ thus creating a dual impediment to effective storage.

Quantitative disparities between seawater and freshwater systems are striking: CarbFix achieved 95% mineralization within 2 years using freshwater,^15^ while seawater experiments consistently yielded <20% over comparable time scales in batch^35,40^ and flow-through (current study) conditions. Wolff-Boenisch and Galeczka^36^ required artificial supersaturation to achieve carbonate precipitation in seawater at 90 °C, a condition rarely sustained in natural systems.

The presence of sulfate in seawater suppresses carbonate nucleation and growth through calcium-sulfate complexation and surface adsorption, resulting in nucleation-limited kinetics despite supersaturated conditions. SEM-EDS imaging revealed no evidence of anhydrite (CaSO_4_) precipitation or other sulfate-bearing phases, indicating that sulfate acted purely as an inhibitor in our system rather than forming competing mineral precipitates.

Despite these limitations, engineering strategies can partially overcome the constraints imposed by seawater. Weak acid coinjection can maintain a favorable pH while preventing excessive clay formation.^36,62^ Alternatively, hybrid approaches that use initial freshwater slugs to establish carbonate precipitation zones, followed by seawater injection, may optimize both water use and mineralization efficiency. Temperature optimization around 90–130 °C balances enhanced kinetics against anhydrite precipitation risks,^35^ though careful monitoring remains essential to prevent near-wellbore permeability loss.

Seawater serves as the primary fluid for offshore basaltic storage, aside from supercritical fluid injection, where containment is assured. Solubility trapping offers a reliable mechanism for intermediate-term storage security, enhancing CO_2_ sequestration in the dissolved phase, while mineralization advances gradually in diffusion-dominated zones. The slower mineralization rates are mitigated by the extensive storage capacity of basaltic reservoirs, which feature dual-porosity systems comprising vesicular networks and fractured matrices. These systems sustain favorable injectivity despite partial mineral precipitation. Through effective pressure management and reservoir engineering, CO_2_ storage operations can accommodate prolonged storage operation time scales without sacrificing injection efficiency, enabling permanent carbon fixation over decades.

#### 4.5.4 Limitations

We recognize that the packed granular bed employed in our flow-through experiments exhibits a higher average coordination number and a more uniform macroscopic flow distribution compared to the low-coordination, often chain-like vesicular networks characteristic of natural basaltic reservoirs. In principle, these differences in pore topology and hydrodynamic regime could modulate the degree of stochasticity observed in reactive transport processes. Nevertheless, the primary experimental evidence supporting nucleation-controlled, patchy precipitation derives from pore- to grain-scale observations. These observations are fundamentally rooted in the probabilistic character of heterogeneous nucleation events and are governed by local interfacial and hydrodynamic heterogeneities—features that are expected to persist, and indeed may be amplified, within vesicular structures with larger stagnation zones and greater pore–substrate heterogeneity. Accordingly, the pore-scale analyses and PNM results presented here should be interpreted as scenario analyses rather than the direct upscaling of the column experiments. They leverage the observed qualitative site-preference patterns to construct physically informed stochastic precipitation scenarios while systematically exploring the topological vulnerability under low-coordination conditions typical of natural vesicular systems.

Powder XRD analysis of representative reacted samples showed no detectable new mineral phases (carbonates or smectite-like clays) relative to pristine basalt glass, consistent with the very small volume fraction of surface precipitates relative to the bulk substrate. This volume-averaged limitation of XRD analysis precludes quantification or even clear detection of thin surface layers, so phase identifications remain inferred from the SEM–EDS morphology and elemental signatures, with explicit acknowledgment of potential amorphous or mixed phases.

A principal limitation of the 1D PHREEQC reactive transport model is its inability to capture the stochastic, highly localized nature of carbonate nucleation and precipitation observed at the pore and grain scales in our experiments. As a deterministic, continuum-scale approach, it assumes laterally uniform reaction progress within each computational cell and does not explicitly account for critical pore-scale heterogeneities that control site-specific nucleation, including surface energy barriers, local hydrodynamic microenvironments (e.g., stagnation zones, substrate surface features, dead-end pores), intrinsic variability in surface reactivity across basalt glass grains, and pore-space geometries. Consequently, the model faithfully reproduces the observed effluent chemistry, glass dissolution rates, and column-scale trends in pH and saturation indices, demonstrating that thermodynamic supersaturation is necessary but insufficient for precipitation. However, it cannot predict the patchy, probabilistic spatial patterns observed in our tests. These pore-scale stochastic features are instead directly evidenced by the experimental results and are mechanistically explored through PNM sensitivity analyses. By combining complementary approaches—such as 1D continuum modeling for bulk geochemical evolution and thermodynamic drivers, PNM for topological vulnerability and precipitation placement implications, and prior LBM work for probabilistic pore-scale dynamics—we obtain a more complete multiscale understanding of the coupled reactive transport and nucleation processes governing CO_2_ mineralization in basaltic systems.

Additional limitations arise from the continuum-scale assumptions and parameter choices in the PHREEQC model. Key boundary conditions (e.g., fixed inlet pCO_2_, open-system CO_2_ equilibration, activity model for seawater ionic strength) and kinetic parameters (basalt glass dissolution rates based on established literature; refer to Section 4.1) were selected to best reproduce the observed effluent chemistry and column-scale pH/saturation trends. No explicit nucleation or inhibition kinetics were included, as the model was neither designed nor capable of simulating pore-scale precipitation patterns. While these choices enable simulation of bulk geochemical evolution and thermodynamic drivers, they inherently limit the model’s ability to capture local heterogeneities or kinetic barriers that govern actual nucleation and spatial distribution.

## 5 Conclusions

This integrated experimental, numerical, and imaging study reveals fundamental controls on CO_2_ mineralization in basaltic systems that challenge conventional reactive transport paradigms.

Carbonate precipitation under reactive flow conditions is a nucleation-controlled probabilistic phenomenon and exhibits pronounced spatial heterogeneity rather than uniform or deterministic patterns. Isolated precipitate pockets form in a patchy, nonuniform distribution along flow paths despite continuous supersaturation, demonstrating that thermodynamic predictions of supersaturation cannot fully forecast actual precipitation locations or timing. Residence time exerts primary control: an order-of-magnitude reduction in flow rate (0.05 to 0.005 mL/min) was necessary for visible carbonate formation, creating spatial partitioning between high-flux, low-mineralization flow highways and low-flux, high-mineralization matrix regions.

Multiscale characterization reveals that vesicular basalts exhibit coordination numbers (modal value = 2), threefold lower than sandstones (modal value = 5), creating a serial rather than parallel flow architecture. Connected porosity fractions (1.3–32.2%) differ dramatically from total porosity (18–42%), emphasizing that topology, rather than total porosity, controls permeability. This low-redundancy architecture renders basalts more vulnerable to permeability loss compared to coordination-6 sandstones.

Secondary mineral assemblages comprise calcium carbonates and inferred smectite-like clays, with magnesium carbonates absent despite thermodynamic favorability, reflecting kinetic limitations below 100 ^°^C. Smectite formation may sequester divalent cations, passivate reactive surfaces, and occlude pore throats, reducing mineralization efficiency but contributing to permanent CO_2_ sequestration through clay-associated trapping.

Seawater use complicates geochemistry, reduces predictability, and likely decreases mineralization rates compared with freshwater systems, though weak-acid injection strategies leveraging solubility trapping may mitigate these limitations with proper engineering.

Successful basaltic CO_2_ storage requires: (1) probabilistic nucleation frameworks in reactive transport models; (2) probabilistic reactive transport modeling to quantify uncertainties in porosity–permeability relations and flow impairment; (3) realistic pore-network topologies from multiscale imaging data; (4) explicit treatment of competing geochemical reactions; (5) adaptive injection management to prevent near-wellbore clogging; and (6) conservative rate estimates accounting for precipitation–permeability coupling. The low-coordination topology of vesicular basalts creates both opportunities (high initial permeability) and vulnerabilities (potential for major permeability loss from modest precipitation), necessitating approaches that are fundamentally different from those used in conventional sandstone reservoir management.
